# Isolation of Phenolic Compounds from Raspberry Based on Molecular Imprinting Techniques and Investigation of Their Anti-Alzheimer’s Disease Properties

**DOI:** 10.3390/molecules27206893

**Published:** 2022-10-14

**Authors:** Qian Wu, Abid Naeem, Jiamei Zou, Chengqun Yu, Yingjie Wang, Jingbin Chen, Yuhui Ping

**Affiliations:** 1 College of Pharmacy, Jiangxi University of Traditional Chinese Medicine, 1688 Meiling Avenue, Nanchang 330004, China; 2 Key Laboratory of Modern Preparation of Traditional Chinese Medicines, Ministry of Education, Jiangxi University of Chinese Medicine, 1688 Meiling Avenue, Nanchang 330004, China; 3 Key Laboratory of Depression Animal Model Based on TCM Syndrome, Jiangxi Administration of Traditional Chinese Medicine, Key Laboratory of TCM for Prevention and Treatment of Brain Diseases with Cognitive Impairment, Jiangxi University of Chinese Medicine, 1688 Meiling Road, Nanchang 330006, China

**Keywords:** UPLC-Q-TOF-MS, molecular imprinting, raspberry, phenolic components, solid phase extraction, molecular docking, neuroinflammation

## Abstract

Alzheimer’s disease is the most common neurodegenerative disease, characterized by memory loss and cognitive dysfunction. Raspberry fruits contain polyphenols which have antioxidant and anti-inflammatory properties. In this study, we used molecular imprinting technology to efficiently isolate phenolic components from the raspberry ethyl acetate extracts. Six phenolic components (ellagic acid, tiliroside, kaempferol-3-o-rutoside, gallic acid, ferulic acid and vanillic acid) were identified by UPLC-Q-TOF-MS analysis. Molecular docking was used to predict the anti-inflammatory effects and anti-Alzheimer’s potential of these isolated compounds, which showed a good binding ability to diseases and related proteins. However, the binding energy and docking fraction of ellagic acid, tiliroside, and kaempferol-3-o-rutoside were better than those of gallic acid, ferulic acid and vanillic acid. Additionally, by studying the effects of these six phenolic components on the LPS-induced secretion of inflammatory mediators in murine microglial (BV2) cells, it was further demonstrated that they were all capable of inhibiting the secretion of NO, IL-6, TNF-α, and IL-1β to a certain extent. However, ellagic acid, tiliroside, and kaempferol-3-o-rutoside have better inhibitory effects compared to others. The results obtained suggest that the phenolic components extracted from ethyl acetate extracts of raspberry by molecularly imprinted polymers have the potential to inhibit the progression of Alzheimer’s disease.

## 1. Introduction

Alzheimer’s disease (AD) is a chronic and progressive neurodegenerative disease that causes memory loss, cognitive impairment, behavioral modifications, and loss of functional abilities. The number of people affected by dementia is expected to increase to 150 million by 2050 [1]. It is probably the most serious economic burden and one of the most common problems in aging societies across the world and is projected to become more serious in the near future. Currently, available drugs can improve AD symptoms to some extent, such as the cholinesterase inhibitor donepezil, memantine, etc. However, the therapeutic effect is not ideal and is accompanied by many side effects [2]. The pathogenesis theories of Alzheimer’s disease include the cholinergic injury theory, AB theory, tau protein theory, neuroinflammation theory, oxidative stress theory, etc. In recent years, neuroinflammation has received increasing attention [3]. A neuroinflammatory model of lipopolysaccharide-induced microglia has become increasingly common in studies of Alzheimer’s disease using BV2 cells, which are mouse glial cells [4,5]. A number of pathological conditions of the central nervous system have been associated with the activation of BV2 cells, which produce NO, tumor necrosis factor (TNF-α), interleukin-6 (IL-6), interleukin-1β (IL-1β) and other cytotoxic molecules, resulting in neuroinflammation. Early detection and inhibition of the release of neuroinflammatory factors in the brain can lead to the successful treatment of AD [6]. Natural products and their biologically active components have become potential candidates as anti-AD agents due to their multi-target properties, which have attracted considerable interest and attention.

Palm leaf raspberry (Rubus chingii Hu) is an immature fruit belonging to the Rubus genus and Rosaceae family. It is an important Chinese herbal medicine serving as a medicine and food with high nutritional and medicinal value [7]. It was also included in the Chinese Pharmacopeia. Raspberry is commonly used in traditional Chinese herbal medicine as a tonic to treat enuresis, impotence, kidney deficiency, frequent urination, and other conditions. It has many pharmacological effects, such as anti-cancer, antioxidant, anti-inflammatory, and memory improvement. In recent years, some scholars have conducted in-depth research on the pharmacological activities of raspberry and found that it also has anti-aging, antianxiety, anti-AD and memory-enhancing properties [8]. Many studies have found that raspberry polyphenols have good effects on cardiovascular disease, oxidative stress, and inflammatory reactions [9,10]. However, raspberry polyphenols have not been extensively studied for Alzheimer’s disease treatment.

Molecular imprinting technology (MIT) uses special target compounds as template molecules to prepare polymers that selectively recognize such target compounds and their structurally similar analogs. It has the advantages of high separation efficiency, stable physical and chemical properties, and easy preparation. At present, molecular imprinting technology has been widely used in the analysis of trace substances in the environment, chemical sensing, separation science, and so on [11]. Furthermore, molecular imprinting technology is applied to extract and separate components of Chinese medicine, for example, using solid phase extraction (SPE) technique for extracting or isolating important compounds from plant extracts. It has the advantages of high extraction efficiency and good selectivity over traditional SPE adsorption materials (such as the C-8 column and the C-18 column) [12]. It can selectively adsorb a class of compounds with the same chemical structure. Currently, the commonly used molecularly imprinted polymer preparation methods include bulk polymerization, in situ polymerization, precipitation polymerization, suspension polymerization, silica gel surface polymerization, and electropolymerization [13]. The surface polymerization method allows better contact between the target molecule and the active site and can also be adapted to meet the needs of various applications by adjusting the particle properties [14].

Structure-based drug design uses molecular docking to predict the conformation of small molecular ligands that will bind to the appropriate binding sites on target molecules. In addition to rational drug design, characterization of binding behavior enables an understanding of the fundamental mechanisms of biological processes. In recent years, molecular docking has become increasingly important in computer-aided drug research [15]. Auto dock vina [16] and discovery studio software are widely used for the molecular docking of compounds with receptors on targeted molecules. The semi-flexible docking method is employed, and the binding energy and docking scores are used to evaluate the docking results.

The current version of the manuscript has been significantly improved from the preprint version [17]. In this study, ellagic acid molecularly imprinted polymer was synthesized by molecular imprinting technology, and its structure, physicochemical and adsorption properties were characterized. Moreover, six phenolic components were isolated from raspberry ethyl acetate extract using the synthesized ellagic acid imprinted polymer and identified by UPLC-Q-TOF-MS analysis. Additionally, a molecular docking study was conducted to determine the affinity for these six components by binding them to inflammatory mediators and AD target proteins. Finally, we verified the effects of the six components on LPS-induced secretion of inflammatory mediators in BV2 cells in order to establish that the raspberry phenolic compounds have therapeutic potential for Alzheimer’s disease treatment.

## 2. Results

### 2.1. Optimization of Synthesis Conditions for Molecularly Imprinted Polymers

#### 2.1.1. Optimization of the Volume of the Pore-Forming Solvent (Carbon Tetrachloride) Volume

Based on the experimental results of Ding Yanhua and others [18], we chose the amount of phenolic components, such as ellagic acid, that is specifically absorbed by the imprinted polymer on the surface of ellagic acid silica gel to be the optimization index. The effects of the amount of carbon tetrachloride, microwave time and microwave power on the synthesis of ellagic acid molecularly imprinted polymer were investigated. Then according to the orthogonal experimental results, carbon tetrachloride volume of 20, 30 and 40 mL, microwave time of 60, 90 and 120 min, and microwave power of 100, 200 and 300 W were selected as the optimization parameters. During the preparation of molecularly imprinted polymers, the volume of carbon tetrachloride (20, 30, and 40 mL) was optimized by maintaining the molar ratios of the template molecule, the functional monomer and the crosslinking agent (1:6:10). The results show that when the carbon tetrachloride volume was 20 mL, a light-yellow polymer with high hardness was obtained. However, when the carbon tetrachloride volume was 30 mL, the light-yellow polymer obtained was milky and uniform in texture, while at 40 mL volume, the reaction did not occur. Therefore, 30 mL of carbon tetrachloride volume was selected.

#### 2.1.2. Optimization of Response Time

The reaction time was optimized by keeping the factors constant while changing the reaction time. The experiments were conducted at 60, 90 and 120 min. In each case, the molecular polymer adsorption capacities were 13.450, 16.392 and 16.290 µmol/g, respectively, so the reaction time of 90 min was selected.

#### 2.1.3. Optimization of Microwave Power

The power of the microwave extractor was optimized by keeping other factors unchanged in the polymerization reaction. The experiments were carried out at 100, 200 and 300 W power, respectively. The reaction power of 100 W does not result in any reaction at the end of the reaction period. When the reaction power is 200 W, a light-yellow milky polymer with a uniform texture appears during the reaction time. In the case of 300 W reaction power, bumping occurs even after the addition of zeolite, but still, no obvious polymer was formed. Therefore, the reaction power of 200 W was selected due to better results.

### 2.2. Characterization of the Polymer Structure

#### 2.2.1. FTIR Analysis

**Figure 1:**
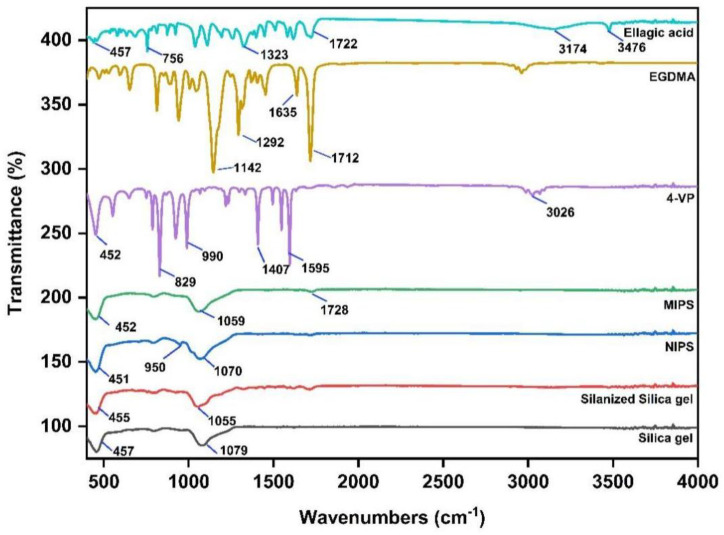
The FTIR spectra of silica gel, silanized silica gel, 4-VP, EGDMA, EA, MIPS and NIPS.

The FTIR spectra of silica gel, silanized silica gel, 4-VP, EGDMA, ellagic acid, MIPs and NPIS were recorded to confirm the synthesis of the product. The results are shown in Figure 1. Silica gel (Si-O-Si) usually exhibits a tensile vibration at 1079 cm^−1^ and a symmetric stretching vibration, and a bending vibration of the Si-O bond at 457 cm^−1^. However, the absorption peak of the silanized silica gel is enhanced at 1055 cm^−1^, and the stretching vibration peak of 1715 cm^−1^ C=O appears, indicating that silanization of the silica gel has occurred. The C-H out-of-plane bending vibration peak of 4-VP is 829 cm^−1^, and the pyridine ring’s C-N stretching vibration absorption peak is 1595 cm^−1^. The crosslinking agent EGDMA has an unsaturated ester structure and has a stretching vibration peak of C=O at 1712 cm^−1^ and asymmetric stretching vibration peaks of C-O-C at 1142 cm^−1^ and 1292 cm^−1^. Furthermore, 3476 cm^−1^ and 3147 cm^−1^ are characteristic stretching vibration peaks of the ellagic acid OH bond [19]. However, the disappearance of the 3147 cm^−1^ peak in MIPS is due to the hydrogen bond formed between ellagic acid and the functional monomer 4-VP, leading to the weakening or disappearance of the peak. This further indicates that the MIPS synthesis is successful. NIPS does not have an ellagic acid template molecule, so the peak at 3476 cm^−1^ is missing. Meanwhile, the absorption peak of the C=C bond at 950 cm^−1^ in NIPS is more intense.

#### 2.2.2. SEM Analysis

**Figure 2:**
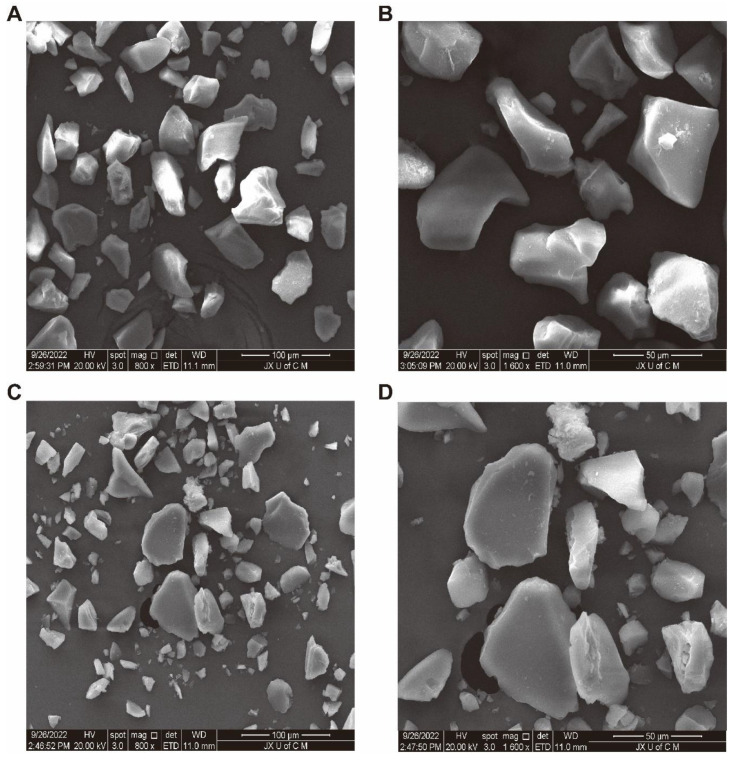
SEM patterns of MIPS at 800x (**A**) and 1600x (**B**), and NIPS at 800x (**C**) and 1600x (**D**).

The scanning electron microscopy results of MIPs and NIPS are shown in Figure 2, At a magnification of 800x, the particle size of MIPS (Figure 2A) is more uniform and larger than that of NIPS (Figure 2C), and the uniformity of the size of NIPS is poor. At the same time, the surface of MIPS (Figure 2B) is more uneven and rougher than that of NIPS (Figure 2D) at 1600x due to the combination with template molecules which makes the polymer surface rougher and favorable for the adsorption of phenolic compounds. This further indicates that the synthesis of ellagic acid-imprinted polymers is successful [20].

#### 2.2.3. XRD Analysis

**Figure 3:**
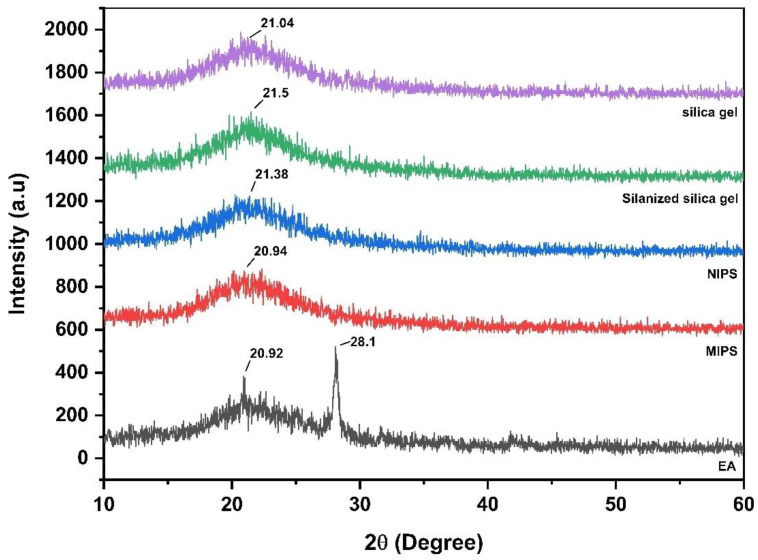
XRD patterns of silica gel, silanized silica gel, MIPs, NIPS, and EA.

XRD was used to study the crystallinity of silica gel, silanized silica gel, MIPs, NIPS and EA powders at room temperature. The XRD results are shown in Figure 3. The diffraction pattern of EA shows numerous sharp characteristic peaks of its crystallinity at 20.92° and 28.1°. These diffraction patterns represent the typical crystal structure of EA. These observations are consistent with previous reports [21]. While the diffraction patterns of silica gel, silanized silica gel, NIPS and MIPS at 2θ have the main broad peaks of 21.04°, 21.5°, 21.38° and 20.94°, respectively. The pattern of these XRD spectra confirms the amorphous structure of these polymers. The 28.1° peak intensity in the range of 2θ in the MIPs diffraction pattern disappears relatively to EA, indicating that the template molecule is complexed with the substrate.

#### 2.2.4. TGA Analysis

**Figure 4:**
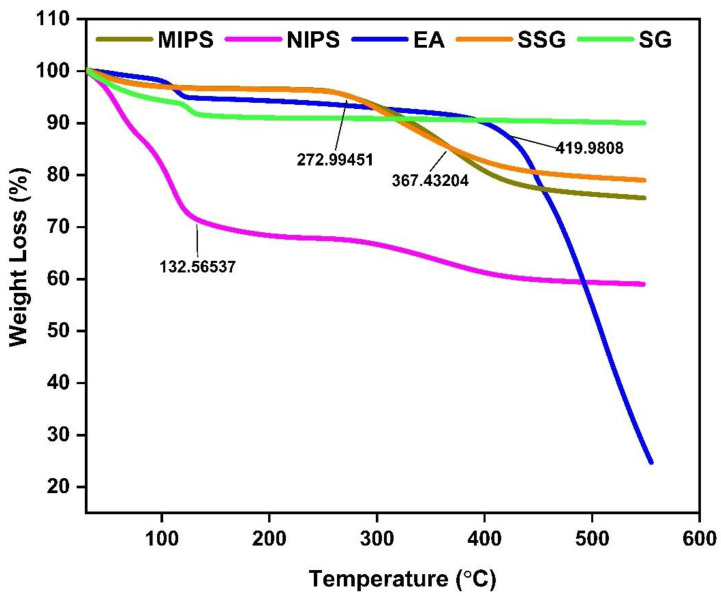
TGA curves of silica gel, silanized silica gel, MIPS, NIPS, and EA.

From the TG curves in Figure 4, At the initial stage, when the mass loss rate is approximately 0.35%, it is due to the decomposition of the crystalline water of the material or the dehydration of the material itself. The MIPS was stable before 272 °C, after which the product began to degrade, and the degradation stopped at 420 °C. This is due to the breakage of the C-C bond in MIPS [20]. Compared to MIPS, the NIPS was significantly degraded at 130 °C. EA loses water rapidly at 100 °C and degrades rapidly at 420 °C, which is caused by the breakdown of hydrogen bonds in EA. The decomposition mode of EA is almost consistent with that reported by previous studies [19]. The weight reduction rate of silanized silica gel at 367 °C is significantly slower than that of silica gel, which can be attributed to the formation of covalent bonds in silanized silica gel which makes it more stable [22].

### 2.3. Adsorption Capacity and Adsorption Kinetics Results

**Figure 5:**
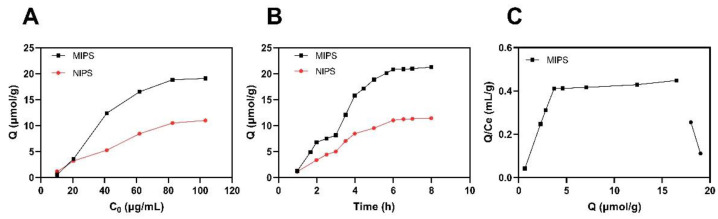
Isothermal adsorption (**A**) and Kinetic adsorption curves (**B**) of MIPS and NIPS and the Scatchard equation of MIPS (**C**).

The adsorption capacity (Q) of the prepared polymers was evaluated by plotting adsorption isotherm curves (Figure 5A). It was shown that the adsorption capacity of MIPS and NIPS increased with increasing the concentration of EA from 10 to 100 mg/L. At about 80 mg/L concentration, the increase in Q for MIPS is faster than for NIPS. The final maximum adsorption capacity of MIPS toward EA is 19.10 μmol/g, which is about two times higher than that of NIPS. These findings indicate that MIPS exhibits a much higher affinity for EA than NIPS [23]. This is because the template molecule leaves “imprinted holes” in the space and structure of the polymer after elution, and specific binding occurs when a molecule with a structure similar to the template molecule enters through the “holes.” The blank imprinted polymer does not contain “holes” similar to the structure of the template molecule.

The results of the analysis of the Scatchard equation are shown in Figure 5C. EA and target molecules are combined in varying ratios to produce different holes with different properties. This is a straight line of non-linear relationships [24]. It indicates that the binding of EA and molecularly imprinted polymers is a two-site or multisite system; the prepared MIPS has more than two affinities, and MIPS may also have positive and negative synergism.

The adsorption kinetics of MIPS and NIPS toward EA were also studied, and the results are shown in Figure 5 B. It should be noted that the equilibrium adsorption time was approximately 6 h when the EA concentration was 60 mg/L, and MIPS exhibits higher adsorption capacity toward EA compared to NIPS. It is apparent that the adsorption process of MIPS increases slowly within 2–3 h, but the increasing speed is obviously accelerated in 3–5 h and gradually comes to equilibrium. The final maximum adsorption capacity of MIPS toward EA is 21.26 μmol/g at 6 h. It indicates that as the binding site on the surface is occupied, it is more and more difficult for template molecules to transfer to the polymer and reach the site, resulting in slow adsorption [25]. It is worth mentioning that the equilibrium time of our presented polymers is relatively long, which can be attributed to the fact that some of the binding sites are located inside the polymeric skeleton.

### 2.4. Qualitative Analysis of the Isolated Compounds by UPLC-Q-TOF-MS

**Figure 6:**
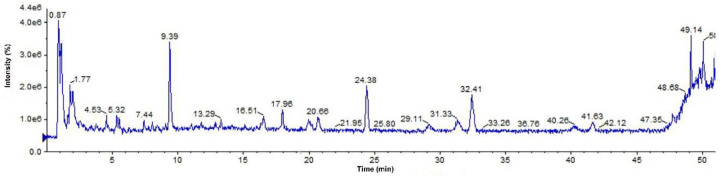
Total ion chromatogram of phenolic components in negative ion mode.

For the analysis and processing of UPLC-Q-TOF-MS data, peak view data software was used, and the names, retention times, molecular formulas, and fragment ions of 6 compounds were obtained, as shown in Table 1. The structures of the compounds were determined on the basis of ion peaks combined with the Mass Bank database and reference structures. The total ion chromatogram in negative ionization mode is shown in Figure 6. A total of six compounds were identified by UPLC-Q-TOF-MS, which guided the subsequent separation and identification of phenolic components in the ethyl acetate fraction of raspberry.

**Figure 7:**
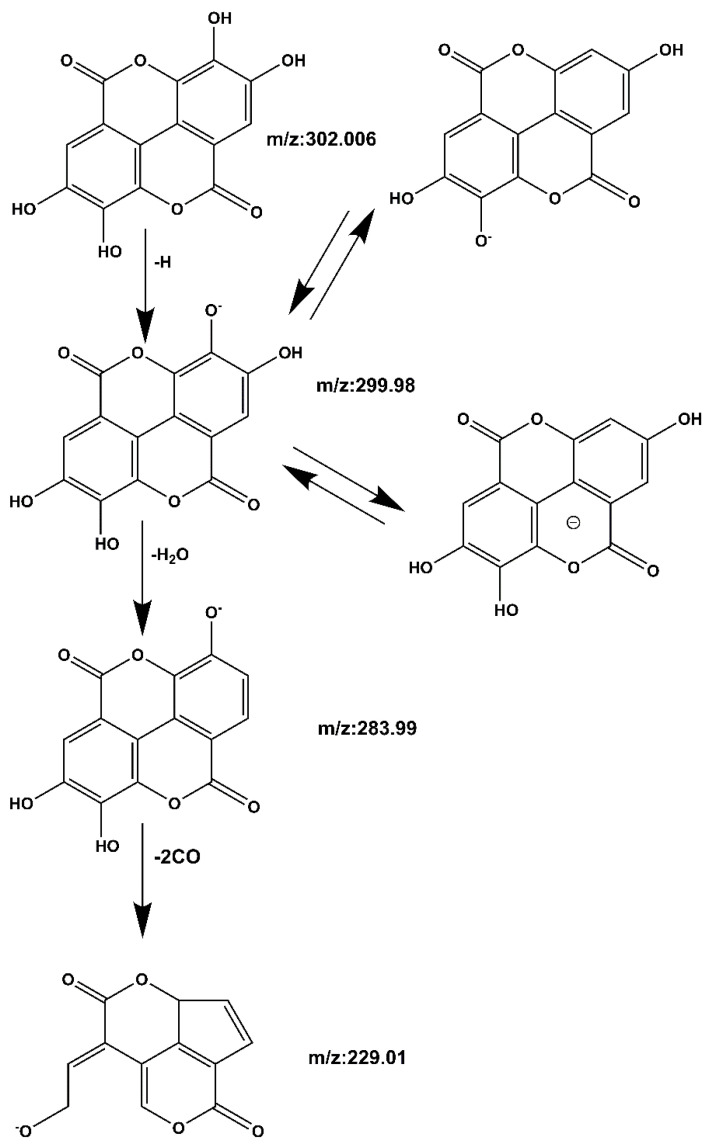
Ellagic acid mass spectrometric cleavage pathway.

**Compound 1 Ellagic acid:** The quasi-molecular ion peak was observed at [M − H]^−^ *m*/*z* 301.006, and the chromatographic retention time was 14.1 min. In the process of fragmentation, the *m*/*z* 302.006 loses one H to produce the fragment *m*/*z* 299.98, then loses one more H_2_O to produce the fragment *m*/*z* 283.99, and loses two more CO to produce the fragment *m*/*z* 229.01. The cracking process is shown in Figure 7. Based on the Mass bank database and comparison with reference material [26,27], it can be inferred that the compound is ellagic acid.

**Figure 8:**
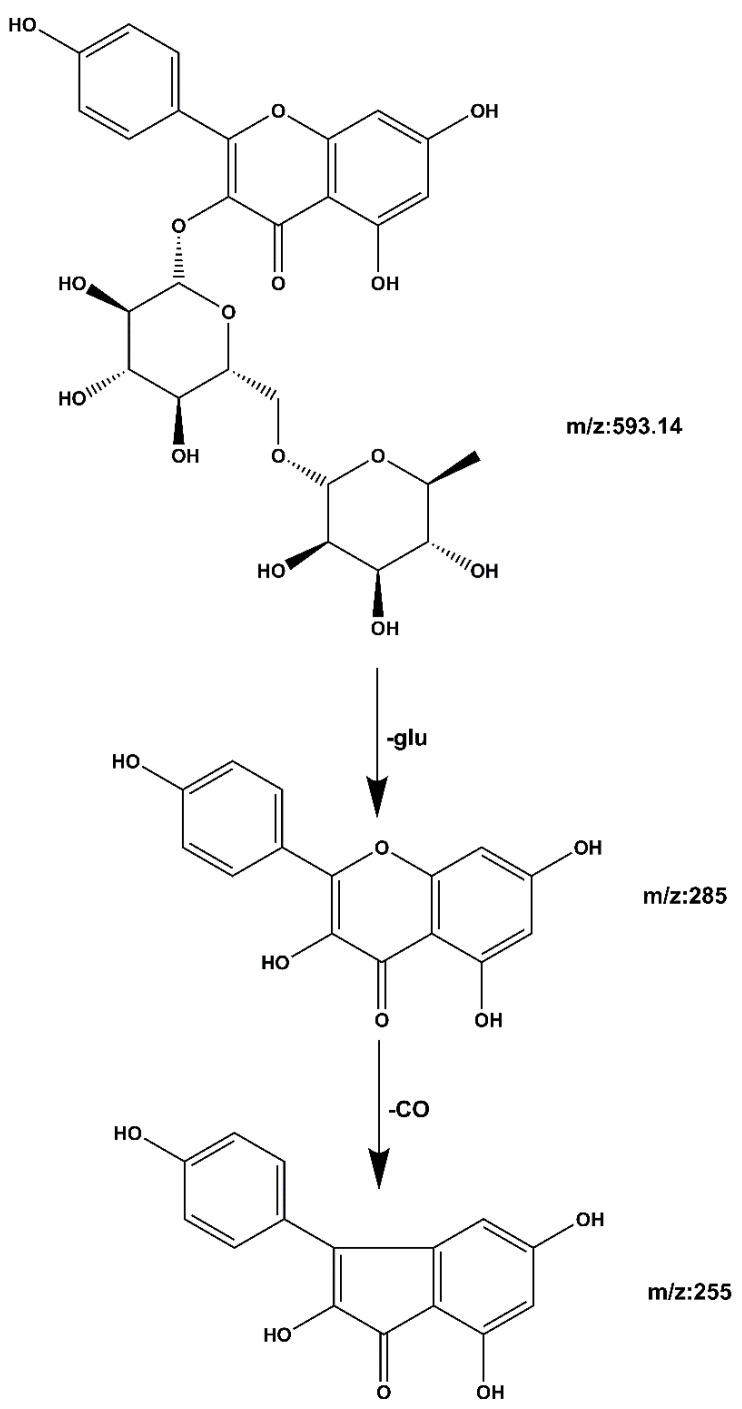
Kaempferol 3-o-rutinoside mass spectrometric cleavage pathway.

**Compound 2 kaempferol 3-o-rutinoside:** The quasi-molecular ion peak was observed at [M − H]^−^ *m*/*z* 593.14 with a retention time of 19.39 min. Base peaks of glycoside compounds are usually formed when the sugar group is removed, among which *m*/*z* 285 is the parent nucleus ion of the aglycone following deglycosylation, and *m/z* 255 is the fragment formed after the removal of the CO group of the C ring. The cracking process is shown in Figure 8. When compared with the literature [28,29], it can be speculated that the compound is kaempferol 3-o-rutinoside.

**Figure 9:**
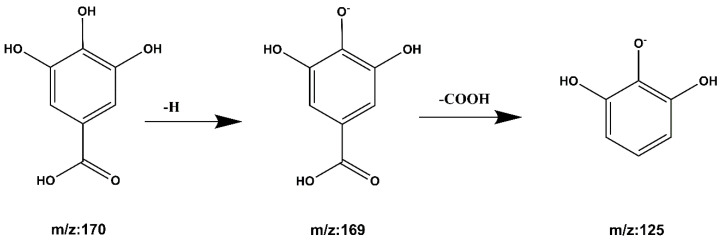
Gallic acid cleavage pathway by mass spectrometry.

**Compound 3 Gallic acid:** The quasi-molecular ion peak is shown at [M − H]^−^ *m*/*z* 169, and the chromatographic retention time is 2.16 min. While *m*/*z* 125 is the ion peak of missing -COOH. The cracking process is shown in Figure 9. Compared with the literature [27,30], it is inferred that the compound is gallic acid.

**Figure 10:**
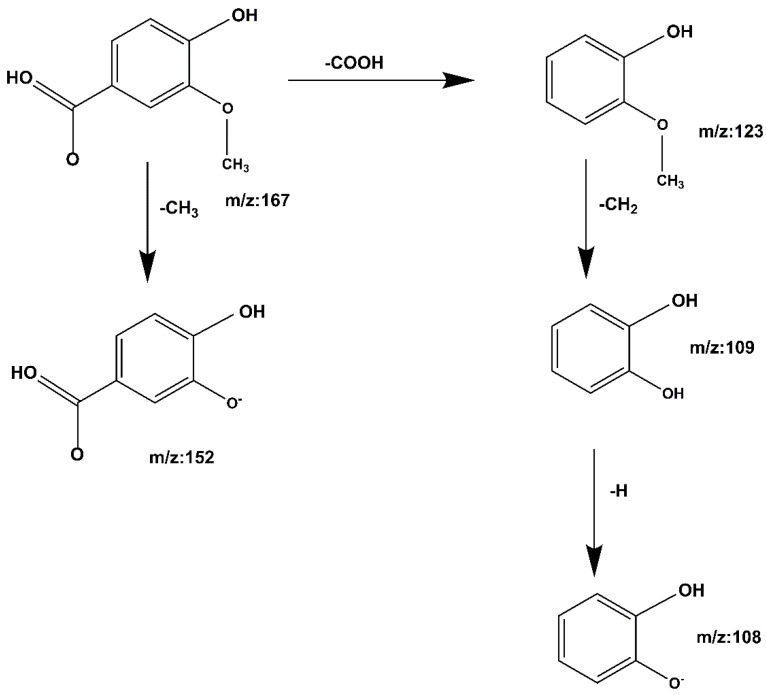
Vanillic acid cleavage pathway demonstrated by mass spectrometry.

**Compound 4 Vanillic acid:** The quasi-molecular ion peak was detected at [M − H]^−^ *m*/*z* 167 with a retention time of 11.22 min. After removal of de–COOH and CH_3_, *m*/*z* 167 is fragmented into *m*/*z* 123, -CH_2_ removed at *m*/*z* 152, *m*/*z* 123 produces fragment *m*/*z* 109, loss of -H at *m*/*z* 109 produces fragment *m*/*z* 108; loss of -COOH at *m*/*z* 152 produces fragment *m*/*z* 108. The cracking process is shown in Figure 10. When compared to the Mass Bank database and reference papers [31,32], it is speculated that the compound is vanillic acid.

**Figure 11:**
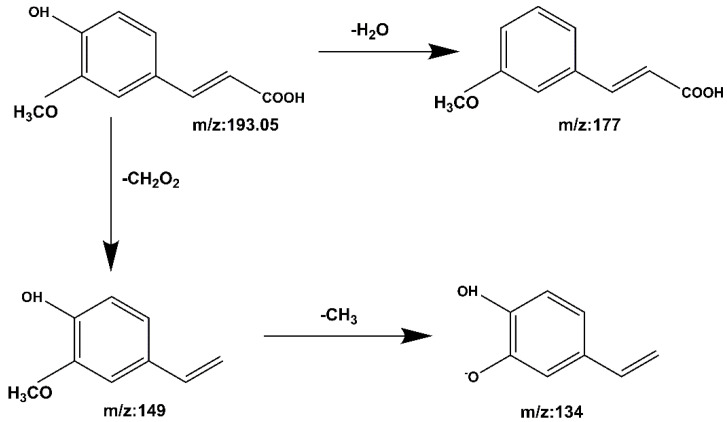
The mass spectrometry cleavage pathway of ferulic acid.

**Compound 5 Ferulic acid:** The quasi-molecular ion peak was detected at [M − H]^−^ *m*/*z* 193.05, and the chromatographic retention time was 13.4 min. Consequently, *m*/*z* 193.05 produces fragments *m*/*z* 177 and *m*/*z* 149 after the removal of H_2_O and CH_2_O_2_. The loss of CH_3_ at *m*/*z* 149 results in fragment *m*/*z* 134. The cracking process is shown in Figure 11. It was identified as ferulic acid by comparing it to the Mass Bank database and the reference literature [31,33].

**Figure 12:**
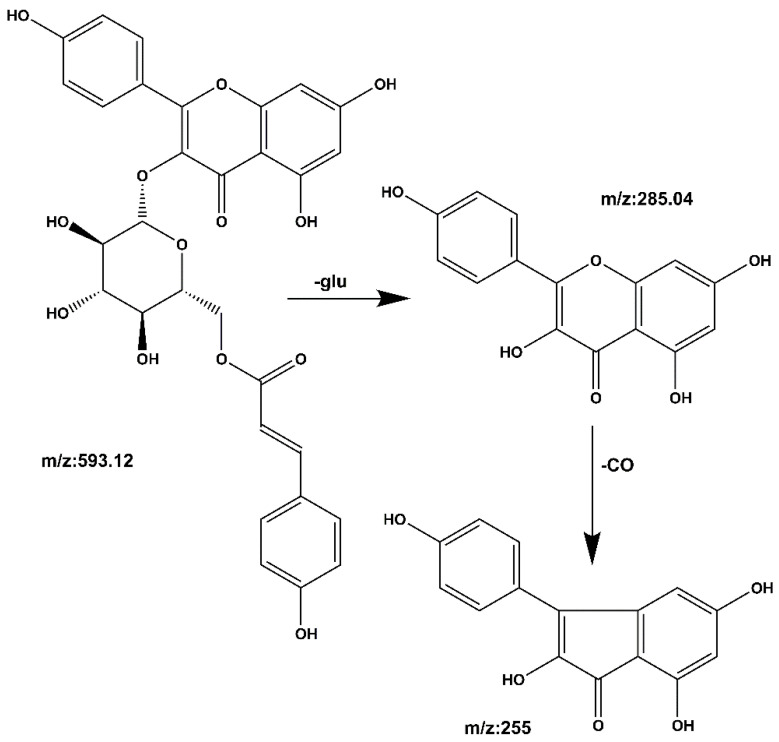
Tiliroside cleavage pathway by mass spectrometry.

**Compound 6 Tiliroside:** The quasi-molecular ion peak was found at [M − H]^−^ *m*/*z* 593.12, with a retention time of 48.15 min. The base peaks of glycosides are usually fragmented after the sugar group has been removed, and the *m*/*z* 285.04 peak results from the removal of the glycosyl group. The aglycon nucleus ion, *m*/*z* 255, is a fragment formed by removing CO from the C ring. The cracking process is shown in Figure 12. It was compared with the reference peaks [33,34] and identified as tiliroside.

### 2.5. Molecular Docking Results

#### 2.5.1. Molecular Docking Results of Six Phenolic Components with TNF-α, IL-1β, and IL-6 Target Proteins

**Figure 13:**
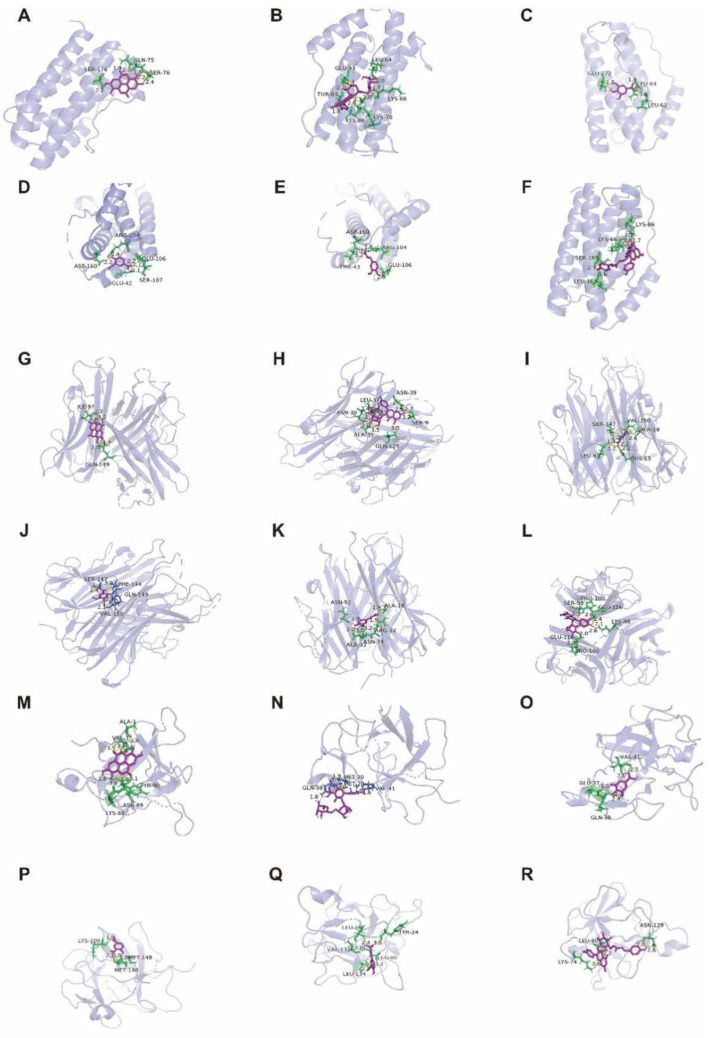
Molecular docking diagram of Ellagic acid (**A**: EA), Kaempferol-3-o-rutin glycoside (**B**: KA), Gallic acid (**C**: GA), Vanillic acid (**D**: VA), Ferulic acid (**E**: FA), and Tiliroside (**F**: TI) with IL-6 (**A**–**F**). Ellagic acid (**G**: EA), Kaempferol-3-o-rutin glycoside (**H**: KA), Gallic acid (**I**: GA), Vanillic acid (**J**: VA), Ferulic acid (**K**: FA), and Tiliroside (**L**: TI) with TNF-α (**G**–**L**), and Ellagic acid (**M**: EA), Kaempferol-3-o-rutin glycoside (**N**: KA), Gallic acid (**O**: GA), Vanillic acid (**P**: VA), Ferulic acid (**Q**: FA), and Tiliroside (**R**: TI) with IL-1β (**M**–**R**) target proteins.

Molecular docking is a computational technique that simulates the interaction between small molecules called ligands and large molecules called receptor proteins. We can predict their affinity by calculating the binding energy between the corresponding molecules after docking using Auto Dock software. The low binding energy (<0) indicates that the two molecules bind spontaneously, and the smaller binding energy means a more stable conformation [35]. The results showed that these six phenolic components could effectively bind to target proteins (Table 2). The binding energies of ellagic acid, tiliroside and kaempferol-3-o-rutoside were low; therefore, it can be predicted that they can better inhibit the release of TNF- α, IL-1β, and IL-6 inflammatory factors. These six active ingredients can interact well with TNF- α, IL-1β, and IL-6. Figure 13 illustrates several docking structures and docking sites in more detail.

#### 2.5.2. Molecular Docking of Six Phenolic Components with Alzheimer’s Disease Targets

We docked the six phenolic small molecules with AD target proteins and then searched for compounds that displayed docking scores greater than 0 and showed the capability of interacting with five or more target proteins simultaneously (Table S1). These compounds were considered potential active compounds. These six compounds have high matching, and the minimum docking score for docking with the target protein was 55.33; therefore, they can be considered potentially active compounds [36].

**Figure 14:**
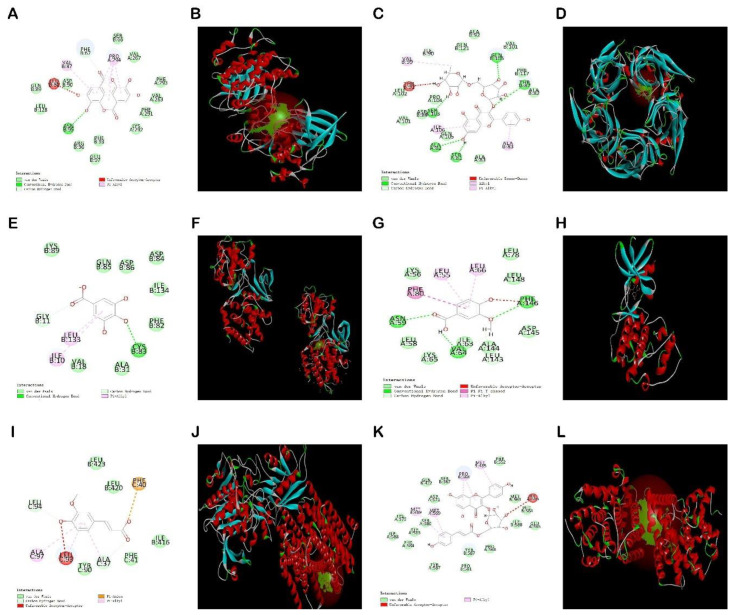
Docking results of six compounds with the highest matching target proteins: EA-GSK3β (**A**,**B**), KA-α7nAchR (**C**,**D**), GA-CDK5 (**E**,**F**), VA-CDK5 (**G**,**H**), FA-γsecretase (**I**,**J**), TI-PDE4A (**K**,**L**). 2D molecular docking diagrams are represented by (**A**) Ellagic acid, (**C**) Kaempferol-3-o-rutin glycoside, (**E**) Gallic acid, (**G**) Vanillic acid, (**I**) Ferulic acid, and (**K**) Tiliroside; and 3D molecular docking diagram are represented by (**B**) Ellagic acid, (**D**) Kaempferol-3-o-rutin glycoside, (**F**) Gallic acid, (**H**) Vanillic acid, (**J**) Ferulic acid, and (**L**) Tiliroside, respectively.

The results showed that each compound corresponded to multiple different proteins. Ellagic acid was successfully matched with eight target proteins, and the docking score was greater than 70, of which GSK3β had the highest matching degree. Kaempferol-3-o-rutoside was successfully matched with six target proteins, and the docking score was greater than 79, of which the highest matching degree was α7nAChR. Gallic acid was successfully matched with 13 target proteins, and the docking score was greater than 55, of which CDK5 had the highest matching degree. Vanillic acid was successfully matched with 12 target proteins, and the docking score was greater than 55, of which CDK5 had the highest matching degree. Ferulic acid was successfully matched with 15 target proteins, and the docking score was greater than 50, of which the highest matching degree was γsecretase protein. Tiliroside was successfully matched with 10 target proteins, and the docking score was greater than 84, of which PDE4A had the highest matching degree. Figure 14 better shows the docking results of ellagic acid, kaempferol-3-o-rutoside, gallic acid, vanillic acid, ferulic acid and tiliroside with the highest matching target protein. The 2D docking diagram shows that it is mainly combined in the mode of hydrogen bond, hydrocarbon bond, π alkyl bond, and anion, and the 3D docking diagram shows the docking site in more detail.

### 2.6. Effects of the Isolated Six Compounds and LPS on the Secretion of Inflammatory Mediators in BV2 Cells

#### 2.6.1. Effects of the Six Compounds and LPS on the Viability of BV2 Cells

**Figure 15:**
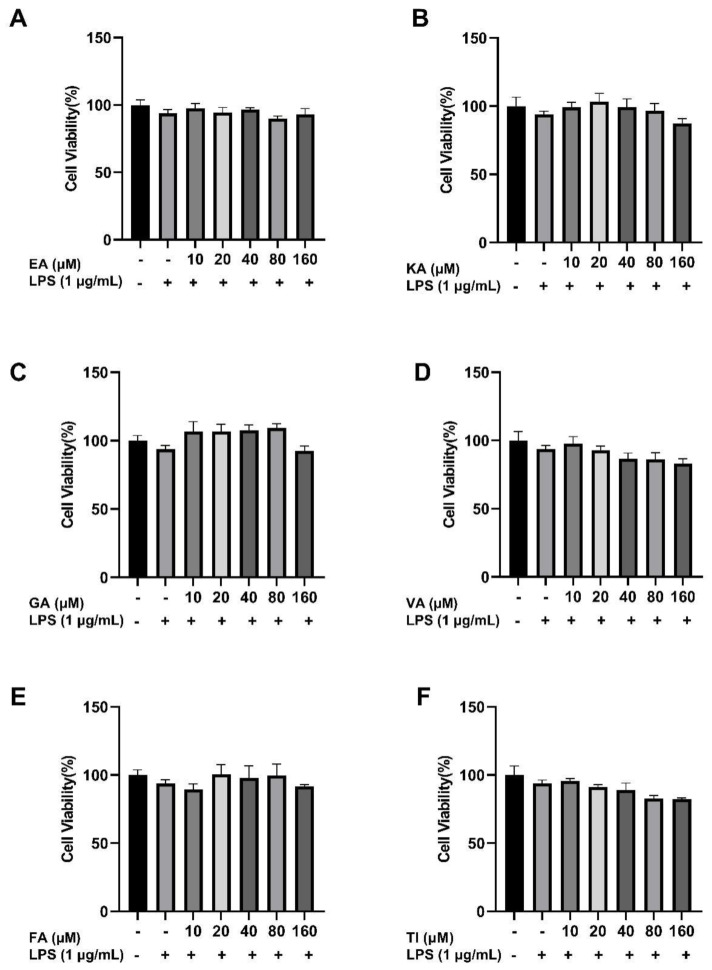
Effect of Ellagic acid (**A**: EA), Kaempferol-3-o-rutin glycoside (**B**: KA), Gallic acid (**C**: GA), Vanillic acid (**D**: VA), Ferulic acid (**E**: FA), and Tiliroside (**F**: TI) at 10, 20, 40, 80 and 160 μM concentrations and LPS on BV2 cell proliferation, respectively. − indicates that contain, + indicates does not contain.

The effects of different concentrations of the six compounds on the viability of BV2 cells were detected by the CCK8 assay. The experimental results are shown in Figure 15 and indicate that the substance did not have significant toxic effects on BV2 cells in the concentration range of 10–160 μM. The survival rate of BV2 cells co-incubated with 1 μg/mL LPS decreased slightly compared to the control group, but there was no significant difference. At the same time, there were no significant differences in the survival rate of these six components when they were co-incubated with LPS with a final concentration of 1 μg/mL in the concentration range of 10–160 μM compared with the control group. According to the published reports [37], it indicates that this concentration range can be used for further experimental research.

#### 2.6.2. Effects of Six Phenolic Compounds on LPS-Induced NO Levels in BV2 Cells

**Figure 16:**
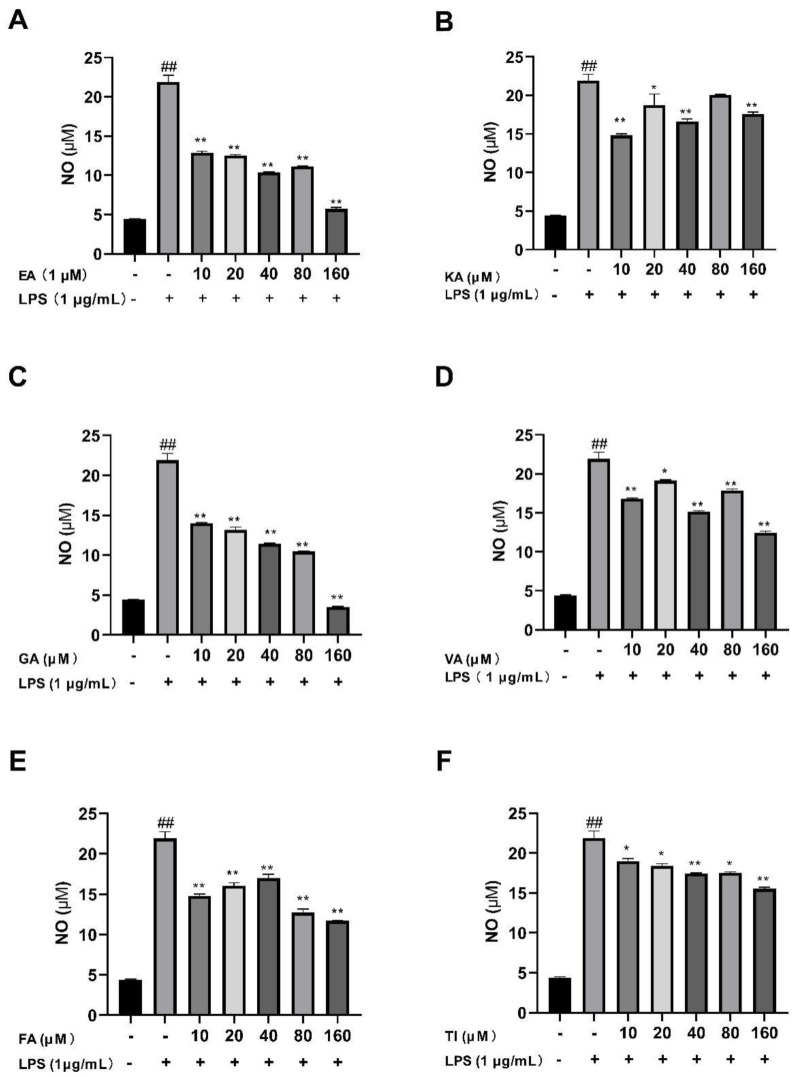
Effects of Ellagic acid (**A**: EA), Kaempferol-3-o-rutin glycoside (**B**: KA), Gallic acid (**C**: GA), Vanillic acid (**D**: VA), Ferulic acid (**E**: FA), and Tiliroside (**F**: TI) on LPS-induced NO release in BV2 cells at 10–160 μM. − indicates that contain, + indicates does not contain. ## represent significant difference between the representative model group and the control group. * represents significant difference compared with model group (*p* < 0.05); and ** represents extremely significant difference compared with model group (*p* < 0.01).

After BV2 cells were stimulated by 1 μg/mL LPS for 24 h, the results showed that the NO levels in the LPS group were significantly higher than that of the control group (##, *p* < 0.01), which indicated that the model was successful as shown in Figure 16 [38]. The drug dose group was treated with prophylactic administration for 4 h before treatment. The results showed that the NO concentration was significantly lower than that of the LPS group in the concentration range of 10–160 μM. The concentration of NO in the ellagic acid, gallic acid, and tiliroside groups decreased with increasing doses, and the trend was good. Furthermore, when the concentration of these six compounds was 160 μM, the NO concentrations decreased significantly in comparison to the LPS group, with an extremely significant difference (*p* < 0.01) [39].

#### 2.6.3. Effects of Six Phenolic Compounds on LPS-Induced TNF-α, IL-1 β, and IL-6 Levels in BV2 Cells

**Figure 17:**
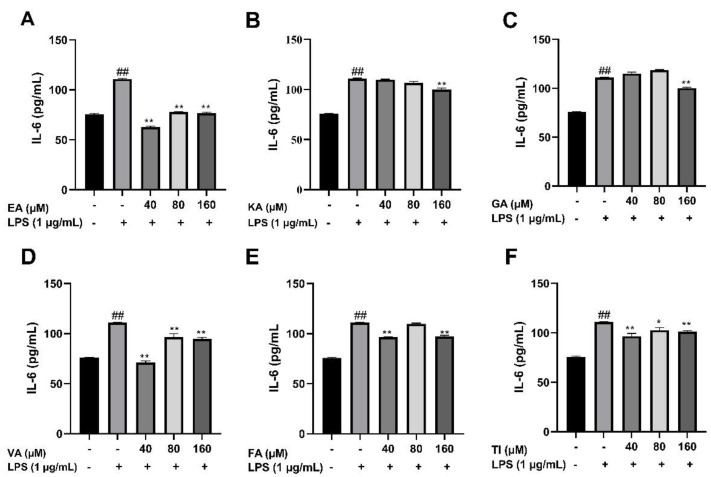
Effects of Ellagic acid (**A**: EA), Kaempferol-3-o-rutin glycoside (**B**: KA), Gallic acid (**C**: GA), Vanillic acid (**D**: VA), Ferulic acid (**E**: FA), and Tiliroside (**F**: TI) on LPS-induced IL-6 in BV2 cells at high (160 μM), medium (80 μM) and low (40 μM) concentrations. − indicates that contain, + indicates does not contain. ## represent significant difference between the representative model group and the control group. * represents significant difference compared with model group (*p* < 0.05); and ** represents extremely significant difference compared with model group (*p* < 0.01).

**Figure 18:**
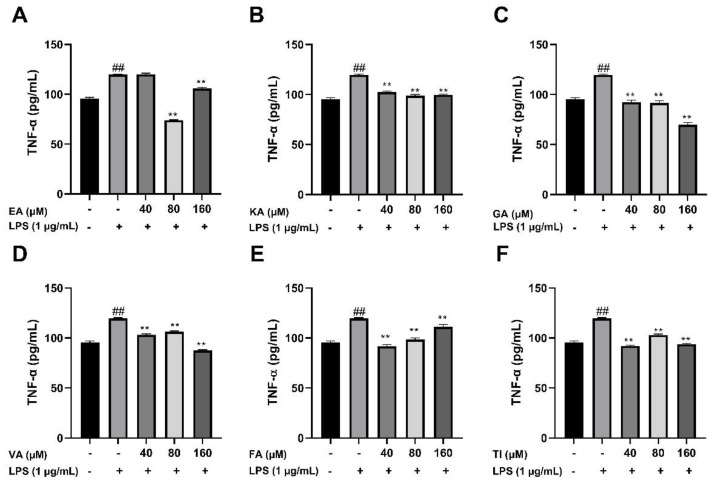
Effects of Ellagic acid (**A**: EA), Kaempferol-3-o-rutin glycoside (**B**: KA), Gallic acid (**C**: GA), Vanillic acid (**D**: VA), Ferulic acid (**E**: FA), and Tiliroside (**F**: TI) on LPS -induced TNF-α in BV2 cells at high (160 μM), medium (80 μM) and low (40 μM) concentrations. − indicates that contain, + indicates does not contain. ## represent significant difference between the representative model group and the control group. ** represents extremely significant difference compared with model group (*p* < 0.01).

**Figure 19:**
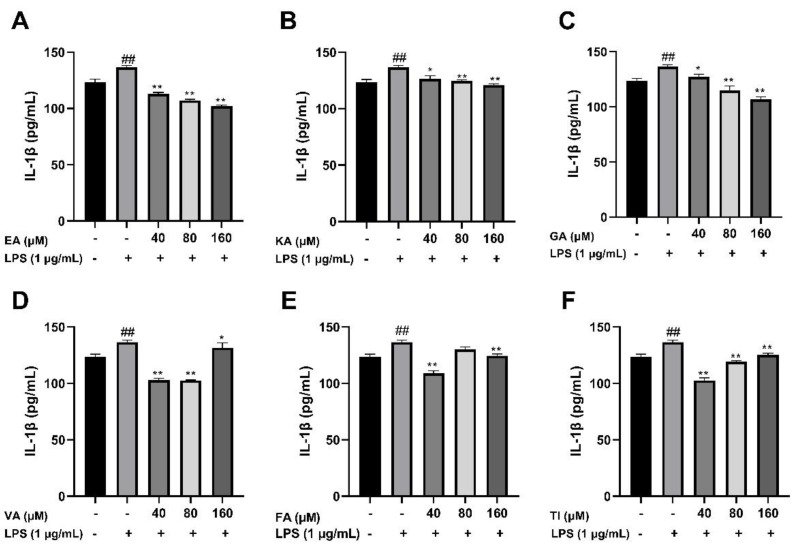
Effects of Ellagic acid (**A**: EA), Kaempferol-3-o-rutin glycoside (**B**: KA), Gallic acid (**C**: GA), Vanillic acid (**D**: VA), Ferulic acid (**E**: FA), and Tiliroside (**F**: TI) on LPS -induced IL-1β in BV2 cells at high (160 μM), medium (80 μM) and low (40 μM) concentrations. − indicates that contain, + indicates does not contain. ## represent significant difference between the representative model group and the control group. * represents significant difference compared with model group (*p* < 0.05); and ** represents extremely significant difference compared with model group (*p* < 0.01).

After BV2 cells were stimulated by 1 μg/mL LPS for 24 h, the cell supernatant was treated with an ELISA kit, and the results showed that the levels of TNF-α, IL-1β, and IL-6 cytokines in the LPS group were significantly higher than that in the control group (##, *p* < 0.01), which indicated that the model was successful. The drug dose group was treated with prophylactic administration for 4 h before treatment, and the experimental results are shown in Figure 17, Figure 18 and Figure 19. The results showed that the levels of inflammatory factors (TNF-α, IL-1β, and IL-6) were significantly decreased at high, medium and low concentrations (160, 80 and 40 μM) of ellagic acid, but the level of TNF-α was not significantly decreased at 40 μM concentrations. At high, medium and low concentrations of kaempferol-3-o-rutoside, the levels of inflammatory factors decreased, and the decrease was more obvious with the increase in dose. At 160 μM, the inhibition effect on the release of inflammatory factors was the best. Coincidentally, the inhibitory effect of gallic acid on the release of inflammatory factors is similar to that of kaempferol-3-o-rutoside. Vanillic acid has a significant inhibitory effect on the release of TNF-α and IL-6 in high, medium and low concentrations (*p* < 0.01). And in medium and low concentrations, it also has an obvious inhibitory effect on the release of IL-1β. However, at 160 μM, the inhibitory effect was not obvious. Ferulic acid significantly inhibited the release of inflammatory factors at high and low concentrations, but the inhibitory effect of the 40 μM concentration was better than 160 μM. The levels of inflammatory factors decreased at high, medium and low concentrations of tiliroside, with a significant difference (*p* < 0.05). Moreover, the inhibition effect of a low concentration of 40 μM was better than that of a high concentration of 160 μM. In general, the inhibitory effects of ellagic acid, tiliroside, and kaempferol-3-o-rutoside were better than those of gallic acid, ferulic acid, and vanillic acid.

## 3. Discussion

Many studies have reported that phenolic compounds have different biological effects on human health, which can combat many chronic diseases, such as cardiovascular diseases, neurodegenerative diseases, and cancer [40]. However, its potential for disease prevention has not been well explored. Ellagic acid is a phenolic component with a high content in raspberry and has multiple biological activities. It has antioxidant, anti-inflammatory, anti-virus, anti-cancer, and other pharmacological activities [41,42]. The traditional separation technology used in traditional Chinese medicine is costly and time-consuming and does not provide the best separation effect. The application of molecular imprinting technology in solid-phase extraction can solve this problem well. It has the advantages of specific adsorption and high separation efficiency and greatly improves the unsatisfactory phenomenon of traditional separation technology [43].

Although traditional methods for the purification of polyphenols, such as liquid chromatography, column chromatography, and high-speed current chromatography, are effective, they are usually labor-intensive and time-consuming. Therefore, it would be advantageous to develop a method for rapidly purifying polyphenols [44,45]. In this study, molecularly imprinted polymer technology was used to fast separate phenolic compounds from raspberry ethyl acetate extracts. Ellagic acid was used as a template molecule to synthesize molecularly imprinted polymers (MIPS) with specific adsorption capacity for phenolic components similar to ellagic acid. Moreover, the ethyl acetate fraction of raspberry has been shown to have an anti-Alzheimer’s effect [46]. And the flavonoid components of raspberry have significantly improved the learning and memory abilities of naturally aging rats and kidney yang deficiency dementia rats, but it is still unknown whether the phenolic components in the ethyl acetate fraction are the main active compounds responsible for this effect or not. Therefore, this study was conducted.

Silica gel, as a carrier in the surface polymerization process, has the characteristics of high adsorption performance, good thermal stability, and stable chemical properties [47]. In the polymer synthesis process, the corresponding synthesis conditions were optimized first, such as the optimal reaction time was 90 min, the optimal microwave synthesis power was 200 W, and the optimal volume of the pore-forming solvent was 30 mL. It is generally consistent with previous studies [48]. The morphology and structure of the synthesized ellagic acid molecularly imprinted polymer were characterized. In the FTIR spectrum, the characteristic C=O stretching band at 1722 cm^−1^ of the EA weakened obviously with the decrease in intensity, and the OH stretching band at 3476 cm^−1^ did not have an absorption peak in the corresponding MIPS spectrum. This may be due to the binding of EA to the substrate as a template molecule. This indicates that the carbonyl and hydroxyl groups of EA participate in the polymerization with MIPS [49]. In the SEM image, MIPs has better size uniformity and better surface smoothness than NIPS under the same magnification, which also shows that the combination with the template molecule (EA) is successful [20]. In the XRD diffraction pattern, the sharp crystal characteristic peaks at 20.92° and 28.1° in the EA diffraction pattern disappear in the MIPS diffraction pattern, further indicating that the template molecule EA has a good combination with the substrate in MIPS [50]. In the TGA analysis, due to binding with the template molecule, MIPS is more stable than NIPS before 420 °C, and it is not easy to lose weight. The silica gel is more stable than the silica gel before 320 °C because silanized covalent bonds in the silica gel make its properties more stable. As reported in the literature [51], dynamic and static adsorption performance tests can effectively detect the adsorption capacity of synthesized MIPS. Whether static adsorption or dynamic adsorption, the adsorption amount Q of MIPS is much larger than that of NIPS because the template molecule EA is eluted after binding to the substrate and leaving imprinted pores. These imprinted pores enable MIPS to selectively adsorb phenolic components with structures similar to those of EA. Scatchard curve analysis showed that two types of binding sites, high-affinity sites and low-affinity sites, were formed on MIPS [52].

Various separation methods, such as silica gel column chromatography and octadecyl silane (ODS) column chromatography, are used for the separation of phenolic compounds from raspberry [53,54]. However, the silica gel column chromatography was able to separate only five compounds, while octadecyl silane column chromatography separated six compounds, which included both phenolics and aldehydes, as a result showing poor selectivity. In this study, the ellagic acid molecularly imprinted polymer was able to successfully separate six phenolic compounds with good specificity. The ellagic acid molecularly imprinted polymer has the characteristics of high adsorption performance and a good separation effect and has high adsorption selectivity for phenolic compounds with a similar structure to the template molecule like ellagic acid. In addition, according to previous studies, MIPS shows excellent regeneration performance, selective adsorption capacity and has good application potential [55].

The synthetic ellagic acid molecularly imprinted polymer was used to extract the ethyl acetate extract of raspberry, and six phenolic components were qualitatively analyzed by UPLC-Q-TOF-MS and were identified as ellagic acid, kaempferol-3-o-rutinoside, gallic acid, vanillic acid, ferulic acid, and tiliroside. Furthermore, molecular docking has been widely used to predict the bindings of small molecules and drugs to their protein targets and to predict their binding affinities and activities [56]. Auto dock software and Discovery Studio software were used to dock the separated six small molecular compounds with IL-6, TNF-α and IL-1β inflammatory factors and AD disease target proteins, respectively. The results showed that these six components could be well connected with it. However, the binding energy and docking fraction of ellagic acid, tiliroside, and kaempferol-3-o-rutoside were better than those of gallic acid, ferulic acid and vanillic acid. This is similar to the results of other researchers [57,58]. The docking results show that ellagic acid has the best docking effect with the GSK-3β target protein, tiliroside has the highest docking score with the PDE4A target protein, kaempferol-3-o-rutoside can dock well with α7nAchR target protein, gallic acid can dock well with the CDK5 target protein, ferulic acid can dock with γsecretase target protein, and vanillic acid can dock well with the CDK5 target protein.

In in vitro cell experiments, a concentration gradient range of 10–160 μM is selected because, within this concentration range, the cell survival rate of BV2 cells is more than 80%, which also indicates that co-culture of these six phenolic components with LPS will not cause toxic effects on BV2 cells [59]. Even in some concentration ranges, it is beneficial for the proliferation of BV2 cells. Furthermore, we detected the concentration of NO released from the supernatant of BV2 cells induced by LPS using the Griess method. It was found that these components inhibited the release of NO well compared with the LPS group in the concentration range of 10–160 μM. The ELISA kit was used to detect inflammatory mediators induced by LPS in the supernatant of BV2 cells. The results showed that under the action of high, medium, and low concentrations (160, 80, and 40 μM) of these phenolic components, compared to the LPS group, the concentration of IL-6, TNF-α and IL-1β was significantly inhibited. The inhibitory effect of ellagic acid, kaempferol-3-o-rutoside, and gallic acid on inflammatory mediators was better at high concentrations than at low concentrations. However, the inhibitory effect of vanillic acid, ferulic acid, and tiliroside at low concentrations was better than that at high concentrations. This may be because different concentrations of different drugs have different sensitivities to cell effects and therefore have certain differences in drug efficacy [60]. Overall, these phenolic compounds have protective effects on LPS-induced inflammation in BV2 cells. Coincidentally, the results of cell experiments correspond to the results of molecular docking, and the effects of ellagic acid, tiliroside, and kaempferol-3-o-rutoside are better. This also shows that molecular docking technology can effectively predict the relationship between the active ingredients of drugs and diseases. We believe that the results presented in this paper are of great significance for the application of molecular imprinting technology to the separation of active ingredients from traditional Chinese medicine. The effects of these six compounds on the secretion of inflammatory mediators in BV2 cells have a certain reference value for the treatment of neuroinflammation by phenolic ingredients and provide a systematic pharmacological basis for the anti-Alzheimer’s disease effect of phenolic ingredients.

## 4. Materials and Methods

### 4.1. Chemicals and Reagents

Ethylene glycol dimethyl methacrylate, 4-ethylene pyridine, and silane coupling agent KH-570 were purchased from Aladdin Reagent Co., Ltd. (Shanghai, China). Azo diisobutyronitrile was acquired from the Tianjin Guangfu Fine Chemical Research Institute (Tianjin, China). Acrylamide (AM) was purchased from Shanpu Chemical Co., Ltd. (Shanghai, China). The silica gel (30 to 40 μm) was supplied by Dingkang Silica Gel Co., Ltd. (Qingdao, China). Six phenolic compounds (ellagic acid, kaempferol-3-o-rutinoside, gallic acid, vanillic acid, ferulic acid and tiliroside) were purchased from ERFA Biotechnology Co., Ltd. (Chengdu, China). BV2 cells were purchased from Procell Life Technology Co., Ltd. (Wuhan, China). Lipopolysaccharide, 0.25% trypsin-EDTA solution, and high glucose medium DMEM were obtained from Solarbio Co., Ltd. (Beijing, China). Fetal bovine serum was procured from Procell Company (America). PBS was supplied by Biochem Co., Ltd. (Shenzhen, China). The CCK8 reagent test kit was purchased from KGI Bio (Suzhou, China). The ELISA kit was purchased from Enzyme Immunoassay Industrial Co., Ltd. (Suzhou, China).

### 4.2. Instruments

The following instruments were used in this study: Microwave extractor (Xinyi Microwave Chemical Technology Co., Ltd. Shanghai, China), Sartorius BS214-D Balance (Sedris Scientific Instruments Co., Ltd., Beijing, China), KQ3200 ultrasonic cleaner (Kunshan Ultrasonic instrument Factory, Suzhou, China), Automatic triple water distiller (Yarong Biochemical Instrument Co., Ltd., Shanghai, China), Uv-1750 UV spectrophotometer (Shimazu Technology Co., Ltd., Beijing, China), Soxhlet extractor (Nanchang University Glass Instrument Factory, Nanchang, China), GT10-1 high-speed desktop centrifuge (Beijing Times Beili Centrifuge Co., Ltd., Beijing, China), Quanta 250 scanning electron microscope (Oregon, USA), Triple TOF5600 + mass spectrometer, Shimadzu Corporation (Jingdu, Japan), multi-function microplate reader from Biotek (Winooski, VT, USA), X-ray diffractometer (TD-3500 X-ray diffractometer, Shanghai, China), Thermogravimetric analyzer (TG/DTA6300, Seiko, Jingdu, Japan), FT-IR spectrometer (Spectrum Two Platinum spectrometer, Elmer, Waltham, MA, USA), ultra-clean workbench from purification company (Suzhou, China), and inverted microscope from Leica (Wetzlar, Germany).

### 4.3. Synthesis of Ellagic Acid Imprinted Polymers by Silica Gel Surface Polymerization

#### 4.3.1. Silica Gel Acid Activation

Silica gel (100 g) was activated by adding 50% concentrated hydrochloric acid (*v*/*v*), and the solution was then refluxed for six hours, cooled, washed with distilled water, vacuum dried for 12 h at 80 °C, and stored in a brown reagent bottle for later use.

#### 4.3.2. Silanization of the Silica Gel Surface

During this experiment, 50 g of acid-activated silica gel and 20 mg of silanization reagent were added to 250 mL of anhydrous toluene. A reaction temperature of 95 °C was maintained, a stirring speed of 300 rpm was used, a microwave was used for 2 h under nitrogen protection, and a microwave power of 100 watts was used to reflux the reaction for a specified period of time. Then it was filtered, transferred to a Soxhlet extractor, and extracted with absolute ethanol for 12 h to remove impurities. Finally, it was vacuum dried at 80 °C for 12 h to obtain the silanized silica gel [61].

#### 4.3.3. Preparation of Ellagic Acid Imprinted Polymer on the Silica Gel Surface

The presence of high levels of active phenolics in the ethyl acetate fraction of raspberry gives ellagic acid a significant advantage over other components as template molecules [62]. Several in vitro and in vivo studies have demonstrated the anti-cancer, anti-viral, anti-oxidant, and anti-chronic disease properties of ellagic acid [63]. Therefore, ellagic acid was chosen as a template molecule. The ellagic acid imprinted polymer was prepared by the following procedure. Briefly, ellagic acid (40 mg) was completely dissolved in 10 mL of dimethyl sulfoxide, and then the surface silanized silica gel (5 g) was added, stirred and mixed at room temperature. Then 4-vinyl pyridine (54 μL), ethylene glycol dimethacrylate (225 μL), and azobisisobutyronitrile (39 mg) were added. Finally, it was transferred to a three-neck flask containing 30 mL of carbon tetrachloride. During this process, a microwave extractor with a power output of 100 watts was used. The reaction was refluxed at 60 °C for 1.5 h, followed by extraction with methanol and acetic acid (9:1, v/v) in a Soxhlet extractor in order to remove template molecules from the polymer. Afterward, it was centrifuged at 5000 rpm until no template molecules were detected in the supernatant. Lastly, it was neutralized with methanol in order to obtain MIPS. The final product was dried in a vacuum oven at 60 °C before use [64]. The blank molecularly imprinted polymer (NIPS) was prepared in the same way as above; however, no template molecule was added.

### 4.4. Characterization of the Polymer Structure

#### 4.4.1. Fourier Transform Infrared Spectroscopy (FTIR)

The vacuum-dried molecularly imprinted polymer, template molecule (EA), substrate (4-VP), and crosslinking agent (EGDMA) were evaluated by Fourier transform infrared spectroscopy. All FTIR spectra were collected in the spectral region of 4000–400 cm^−1^.

#### 4.4.2. Scanning Electron Microscope (SEM)

Surface topology differences between MIPS and NIPS were compared by high-resolution scanning electron microscopy [65].

#### 4.4.3. X-ray Diffraction (XRD)

XRD studies were performed on the synthesized polymers and the raw components. The samples were irradiated with monochromatized Cu-Kα radiation and analyzed at the 2θ angle between 10 and 60° with 0.02° increments and a recording time of 2 s. The voltage and current used are 30 kV and 15 mA, respectively.

#### 4.4.4. Thermogravimetric Analysis (TGA)

For TGA analysis, the samples (5–10 mg) were added to a standard alumina crucible and heated from 30 to 600 °C at a constant heating rate of 10 °C/min under nitrogen gas.

### 4.5. Dynamic and Static Adsorption Experiments

Molecularly imprinted and blank imprinted polymers were weighed (25 mg) and added to a flask containing 5 mL of EA standard solution at concentrations of 10, 20, 40, 60, 80 and 100 µg/mL. The polymer was adsorbed at a constant temperature until saturation was reached, then centrifuged, and the supernatant solution was taken and diluted appropriately. After that, the adsorption capacity (Q) was determined on the basis of absorbance using a UV spectrophotometer. Kinetic binding experiments were carried out similarly, except that the EA concentration was kept constant at 60 μg/mL, and the determination was made at different time intervals (1, 2, 3, 4, 5, 6, 7 and 8 h). Then, the adsorption isotherm curve was made based on the Q value and the initial concentration C0. The adsorption capacity (Q) was calculated using Equation (1), where Q is the static equilibrium adsorption capacity (μmol/g), C0 is the initial concentration of template molecules (μmol/L), Cs is the concentration at adsorption equilibrium (μmol/L), V is the volume of the substrate solution (mL), and M is the amount of polymer used (mg).

(1) $$Q=\frac{\left(C_{0}-C_{S}\right)V}{M}$$

In order to better and more intuitively evaluate the adsorption performance of MIPs, the combination of ellagic acid and target molecules is reflected in the Scatchard equation [66]. The Scatchard equation is shown in Equation (2). The equation describes the binding action between receptor and ligand, showing that there is no interaction at the binding site. Where Q is the adsorption amount (μmol/g); Kd is the equilibrium dissociation constant of the binding site; Qmax is the maximum adsorption amount of the binding site (μmol/g), CEA is the equilibrium mass concentration of the substrate in the supernatant (μ mol/L).

(2) $$\frac{Q}{C_{\mathrm{EA}}}=\frac{Q_{\max}-Q}{K_{d}}$$

### 4.6. Solid Phase Extraction from Raspberry (Ethyl Acetate Extract) Using Ellagic Acid-Imprinted Polymers

Raspberry ethyl acetate extract (0.1 g) was dissolved in 10 mL of methanol, and then ellagic acid silica gel surface imprinting polymer (2 g) was added and agitated for 1 h. Subsequently, it was loaded onto a chromatographic column with a diameter of 1.2 cm and a length of 35 cm. A perforated flat sieve plate is fitted at the bottom. Before use, the column is washed repeatedly with ethanol and soaked in acetonitrile. The chromatographic column was pre-balanced with 10 mL methanol. The eluents were acetonitrile and methanol, and the chromatographic column was washed with 10 mL of acetonitrile followed by 10 mL of methanol. After collecting the effluents of the washing and elution steps, the methanol eluent was evaporated to dryness in a nitrogen-blowing water bath, and then methanol was added to the eluent to make up the final volume of 2 mL. This sample, which contains phenolic components, was then analyzed by UPLC-Q-TOF-MS analysis.

### 4.7. Identification of Isolated and Enriched Compounds Based on UPLC-Q-TOF-MS

#### 4.7.1. UPLC-Q-TOF-MS Conditions

Chromatographic conditions: column Waters Symmetry C18 (4.6 mm×250 mm, 5 μm); column temperature: 30 °C; flow rate: 0.2 mL/min; injection volume: 2 μL; mobile phase A: acetonitrile and mobile phase B: 0.1% formic acid aqueous solution; Gradient elution: 0–10 min, 5%–13%; 11–15 min, 13%–15%; 16–25 min, 15%–16%; 26–35 min, 16%–40%; 36–45 min, 17%–18%; and 46–60 min, 40%–40%.

Mass spectrometry conditions: The mass spectrometer equipped with a Duo Spray TM source (AB SCIEX, Vaughan, ON, Canada) was run in a negative ESI mode at a mass range of *m*/*z* 100–1500. The following parameters were set up and used: 500 °C ion source heater; 4500 V ion spray voltage; 40 psi curtain gas; 50 psi ion source gas 1; 50 psi ion source gas 2; and survey scan. In addition, MS/MS investigation was performed with an accumulation time of 250 ms for Q-TOF-MS, the accumulation time was 100 ms for MS/MS analysis, and collision cell inlet voltage was 10 eV, and the collision cell exit voltage was 18 eV. The acquired mass data were imported and analyzed using peak view software (AB Sciex, Foster City, CA, USA). Moreover, information-dependent acquisition (IDA) was employed to trigger the acquisition of MS/MS spectra for ions corresponding to the IDA requirements. The real-time multiple mass defect filter (MMDF) was utilized in the IDA criteria, and priority was given to the ions matching the mass defect window in acquiring MS/MS spectra.

#### 4.7.2. Qualitative Analysis Using UPLC-Q-TOF-MS

Following solid phase separation, 2 mL of phenolic extracts are filtered through a 0.22 μm microporous membrane. Then the filtrate is transferred to a liquid phase vial and analyzed by UPLC-Q-TOF-MS analysis.

### 4.8. Molecular Docking

Molecular docking is a technique that allows us to rapidly identify active compounds that have the effect of treating related diseases from a large number of compounds and carry out relevant pharmacological experiments to determine the mechanism of action of a drug. Potential targets of Alzheimer’s disease were identified by network pharmacology and the disease target screening process combined with the TCMSP database. TNF-α, IL-6, and IL-1β macromolecular and disease target proteins were retrieved from the protein database (http://www.rcsb.org/pdb/ (accessed on 13 September 2022)). The structural formula of 6 compounds was searched using the ChemSpider database (http://www.chemspider.com/ (accessed on 13 September 2022)), downloaded and saved as a mol format. Molecular docking is performed on pretreated target proteins using Auto Dock or Discovery Studio software. The pretreated target proteins are obtained after removing water molecules, polar hydrogen and protein molecules from the PDB file.

Docking screening experiments were performed by removing the co-crystallized ligand and docking the prepared ligands to the protein using the same docking parameters. The binding sites were determined by analyzing the binding of the co-crystallized ligands and the corresponding factors, such as TNF-α, IL-6 and IL-1β. The docking results were analyzed, and the docking model with the lowest docking energy or the highest binding fraction representing the best ligand binding site of the compound was selected. The energy of the interaction was calculated for each ligand to define its interaction with the receptor, and the predicted binding interactions between the ligand and the receptor were examined.

### 4.9. In Vitro Anti-Alzheimer Studies of Isolated and Identified Components

#### 4.9.1. Effects of Isolated Components on the Proliferation of LPS Treated BV2 Cells

BV2 cells were cultured in a high glucose DMEM medium containing 10% fetal bovine serum and 1% antibiotics (penicillin and streptomycin) and placed in a humidified incubator at 37 °C and 5% CO_2_. BV2 cells in the logarithmic growth phase were taken, digested with trypsin, fresh media was added and adjusted the cell density to 1 × 10^5^ cells per mL and seeded in 96-well plates. Cells were divided into four groups after adhering to the wall: blank group, control group, LPS group and drug dose group (10, 20, 40, 80 and 160 μM), with five parallel duplicates for each group. The blank group contained only a medium, whereas the control group was the cells cultured in a conventional medium. After the drug dose group was pretreated with drugs for 4 h, the LPS group and the drug dose group were added 1 μg/mL of LPS treatment for 24 h. The CCK8 solution (10 µL) was then added to each well, avoiding bubbles, and shaken well using the cross method. After 30 min of incubation, the OD value was measured using a microplate reader at 450 nm. Equation (3) was used for the calculation of the cell survival rate per well. Where, As is the absorbance of the experimental well, Ab is the absorbance of the blank well, and Ac is the absorbance of the control well.

(3) $$\mathrm{Cell}\;\mathrm{viability}\left(\%\right)=\frac{\left(\mathrm{As}-\mathrm{Ab}\right)}{\left(\mathrm{Ac}-\mathrm{Ab}\right)}\times 100$$

#### 4.9.2. NO Level Detection Using Griess Method

The cell treatment method is the same as in 2.8.1. After 24 h of culture, the cell supernatant was taken, and Griess reagent was added. The OD value of different components was measured at a wavelength of 540 nm.

#### 4.9.3. Detection of TNF-α, IL-1 β, IL-6 Levels by ELISA

The cell suspension (1 × 10^5^ cells per mL) was seeded in 24-well plates with 500 μL of volume in each well. They were divided into blank group, LPS group, and low-, medium- and high-dose drug groups (40, 80, and 160 μM). After 24 h of treatment with the same method, the cell supernatant was centrifuged at 1000× *g* at 4 °C for 15 min, and then the supernatant was aspirated. Then, it was treated with the solutions provided in the ELISA kit, and the absorbance was measured at 450 nm with a microplate reader. The content of TNF-α, IL-1β and IL-6 were calculated according to the standard curve.

### 4.10. Data Processing and Statistical Analysis

The acquired data was processed through Analyst 1.6 software (AB sciex Corp., Framingham, MA, USA). The compounds were analyzed and identified by Peakview software, and information such as the names, retention times, molecular formulas, and fragment ions of the compounds were retrieved. Statistical experimental analyzes were performed using GraphPad Prism 9.0.1 (GraphPad Software Inc., San Diego, CA, USA), and statistical differences were considered significant if the (*p*-values were < 0.05).

## 5. Conclusions

This study utilized molecular imprinting technology to extract phenolic compounds from the ethyl acetate extract of raspberry that is similar to the template molecule ellagic acid. In addition, UPLC-Q-TOF-MS analyses revealed six phenolic compounds (ellagic acid, kaempferol-3-o-rutinoside, gallic acid, tiliroside, ferulic acid, and vanillic acid) that may be effective in treating Alzheimer’s disease. The binding of these six components to inflammatory mediators (IL-6, TNF-α, IL-1β) and Alzheimer’s disease-related protein targets was studied by molecular docking technology, and it was found that these six components all have low binding energy and good binding fraction. And the docking results of ellagic acid, tiliroside and kaempferol-3-o-rutoside were better. These compounds are both safe and beneficial to the proliferation of BV2 cells at a certain concentration and can effectively inhibit the LPS-induced release of inflammatory factors such as NO, IL-6, TNF-α, and IL-1β of BV2 cells. In general, ellagic acid, tiliroside, and kaempferol-3-o-rutoside have better inhibitory effects. In summary, this study can provide a scientific basis for the development of ellagic acid surface imprinted polymers for solid-phase separation of phenolic compounds, as well as provide a theoretical basis for the treatment of Alzheimer’s disease with phenolic compounds.

## Figures and Tables

**Table 1:** UPLC-Q-TOF-MS ion fragments of six isolated phenolic compounds.

S. No	t_R_	Quality Score	Deviation	Mode	Secondary Fragment Ions	Molecular Formula	Chemical Name
1	14.1	302.006	−2.4	[M − H]^−^	299.9, 283.99, 229.01	C_14_H_6_O_8_	Ellagic acid
2	19.39	594.1585	−4.6	[M − H]^−^	593.1, 285.04, 255.02	C_27_H_30_O_15_	Kaempferol 3-rutinoside
3	2.16	170.0215	4.2	[M − H]^−^	169.0, 125.02, 97.03, 81.04	C_7_H_6_O_5_	Gallic acid
4	11.22	168.0423	2.6	[M − H]^−^	167.0, 152.01, 123.04, 108.02	C_8_H_8_O_4_	Vanillic acid
5	13.4	194.0579	2.6	[M − H]^−^	193.05, 178.0,149.05, 134.03	C_10_H_10_O_4_	Ferulic acid
6	48.15	594.1373	−1.9	[M − H]^−^	593.1, 285.04	C_30_H_26_O_13_	Tiliroside

**Table 2:** The binding energy of six phenolic components to target genes.

Components	Binding Energies (kcal/mol)		
	IL-6	IL-1β	TNF-α
Ferulic acid	−5.1	−5.5	−6.5
Tiliroside	−6.4	−7.8	−9.7
Gallic acid	−5.8	−5.2	−6.5
Ellagic acid	−7	−6.7	−10
Kaempferol-3-o-rutoside	−7.3	−7.3	−8.5
Vanillic acid	−5.6	−4.9	−6.4

## Data Availability

The data is contained within the article.

## Supplementary Materials

The following supporting information can be downloaded at: https://www.mdpi.com/article/10.3390/molecules27206893/s1, Table S1: Molecular docking results of the six phenolic compounds with target proteins of AD.

## Sample Availability

Samples of the compounds are available from the authors upon a reasonable request.

## References

[1] Brookmeyer R., Johnson E., Ziegler-Grahamm K., Arrighi H.M. (2007). O1–02–01: Forecasting the global prevalence and burden of Alzheimer’s disease. Alzheimer’s Dement..

[2] Schneider L.S., Mangialasche F., Andreasen N., Feldman H., Giacobini E., Jones R., Mantua V., Mecocci P., Pani L., Winblad B. (2014). Clinical trials and late-stage drug development for Alzheimer’s disease: An appraisal from 1984 to 2014. J. Intern. Med..

[3] Assadeck H., Daouda M.T., Omar E.A., Mamadou Z., Djibo F.H., Maiga D.D. (2019). Inflammatory demyelinating diseases of the central nervous system in Niger. Rev. Neurol..

[4] Ma H., Johnson S.L., Liu W., DaSilva N.A., Meschwitz S., Dain J.A., Seeram N.P. (2018). Evaluation of polyphenol anthocyanin-enriched extracts of blackberry, black raspberry, blueberry, cranberry, red raspberry, and strawberry for free radical scavenging, reactive carbonyl species trapping, anti-glycation, anti-β-amyloid aggregation, and microglial neuroprotective effects. Int. J. Mol. Sci..

[5] Yirmiya R., Rimmerman N., Reshef R. (2015). Depression as a microglial disease. Trends Neurosci..

[6] Bagyinszky E., Van Giau V., Shim K., Suk K., An S.S.A., Kim S. (2017). Role of inflammatory molecules in the Alzheimer’s disease progression and diagnosis. J. Neurol. Sci..

[7] Sheng J.-Y., Si-Qi W., Kao-Hua L., Bo Z., Zhang Q.-Y., Lu-Ping Q., Jian-Jun W. (2020). Rubus chingii Hu: An overview of botany, traditional uses, phytochemistry, and pharmacology. Chin. J. Nat. Med..

[8] Ali N., Shaoib M., Shah S.W.A., Shah I., Shuaib M. (2017). Pharmacological profile of the aerial parts of Rubus ulmifolius Schott. BMC Complement. Altern. Med..

[9] Burton-Freeman B.M., Sandhu A.K., Edirisinghe I. (2016). Red raspberries and their bioactive polyphenols: Cardiometabolic and neuronal health links. Adv. Nutr..

[10] Chen W., Li Y., Bao T., Gowd V. (2017). Mulberry Fruit Extract Affords Protection against Ethyl Carbamate-Induced Cytotoxicity and Oxidative Stress. Oxidative Med. Cell. Longev..

[11] Vasapollo G., Sole R.D., Mergola L., Lazzoi M.R., Scardino A., Scorrano S., Mele G. (2011). Molecularly imprinted polymers: Present and future prospective. Int. J. Mol. Sci..

[12] Cheong W.J., Yang S.H., Ali F. (2013). Molecular imprinted polymers for separation science: A review of reviews. J. Sep. Sci..

[13] Turiel E., Martín-Esteban A. (2010). Molecularly imprinted polymers for sample preparation: A review. Anal. Chim. Acta.

[14] Xing R., Wang S., Bie Z., He H., Liu Z. (2017). Preparation of molecularly imprinted polymers specific to glycoproteins, glycans and monosaccharides via boronate affinity controllable–oriented surface imprinting. Nat. Protoc..

[15] Morris C.J., Corte D.D. (2021). Using molecular docking and molecular dynamics to investigate protein-ligand interactions. Mod. Phys. Lett. B.

[16] Trott O., Olson A. (2009). Software news and update AutoDock Vina: Improving the speed and accuracy of docking with a new scoring function. Effic. Optim. Multithreading.

[17] Wu Q., Ping Y., Zou J., Huang L., Naeem A., Chen J., Wang Y. Study on the Separation and Cytotoxicity of Raspberry Phenolic Compounds Based on Molecular Imprinting Technology. https://ssrn.com/abstract=4128845.

[18] Xie Y.H., Ding Y.H., Liu W.Q., Bai W.T., Huang L.P. (2015). Microwave assisted synthesis of quercetin-selective MIPs grafted on silica gel surface. Adv. Mater. Res..

[19] Li Y., Zhao X., Zu Y., Zhang Y., Ge Y., Zhong C., Wu W. (2015). Preparation and characterization of micronized ellagic acid using antisolvent precipitation for oral delivery. Int. J. Pharm..

[20] Zhang H., Zhao S., Zhang L., Han B., Yao X., Chen W., Hu Y. (2016). Preparation of ellagic acid molecularly imprinted polymeric microspheres based on distillation–precipitation polymerization for the efficient purification of a crude extract. J. Sep. Sci..

[21] Xiao Y., Zhou L., Hao H., Bao Y., Yin Q., Xie C. (2021). Cocrystals of propylthiouracil and nutraceuticals toward sustained-release: Design, structure analysis, and solid-state characterization. Cryst. Growth Des..

[22] Rekondo A., Irusta L., Fernández-Berridi M. (2010). Characterization of silanized poly (ether-urethane) hybrid systems using thermogravimetric analysis (TG). J. Therm. Anal. Calorim..

[23] Rui C., He J., Li Y., Liang Y., You L., He L., Li K., Zhang S. (2019). Selective extraction and enrichment of aflatoxins from food samples by mesoporous silica FDU-12 supported aflatoxins imprinted polymers based on surface molecularly imprinting technique. Talanta.

[24] Gao C., Liu S., Zhang X., Liu Y., Qiao C., Liu Z. (2016). Two-photon fluorescence and fluorescence imaging of two styryl heterocyclic dyes combined with DNA. Spectrochim. Acta. Part A Mol. Biomol. Spectrosc..

[25] Zeng Y., Zhou Y., Kong L., Zhou T., Shi G. (2013). A novel composite of SiO2-coated graphene oxide and molecularly imprinted polymers for electrochemical sensing dopamine. Biosens. Bioelectron..

[26] Nowicka A., Kucharska A.Z., Sokół-Łętowska A., Fecka I. (2019). Comparison of polyphenol content and antioxidant capacity of strawberry fruit from 90 cultivars of Fragaria× ananassa Duch. Food Chem..

[27] Bursal E., Köksal E., Gülçin İ., Bilsel G., Gören A.C. (2013). Antioxidant activity and polyphenol content of cherry stem (Cerasus avium L.) determined by LC–MS/MS. Food Res. Int..

[28] Abad-García B., Garmón-Lobato S., Berrueta L.A., Gallo B., Vicente F. (2012). On line characterization of 58 phenolic compounds in Citrus fruit juices from Spanish cultivars by high-performance liquid chromatography with photodiode-array detection coupled to electrospray ionization triple quadrupole mass spectrometry. Talanta.

[29] Cheiran K.P., Raimundo V.P., Manfroi V., Anzanello M.J., Kahmann A., Rodrigues E., Frazzon J. (2019). Simultaneous identification of low-molecular weight phenolic and nitrogen compounds in craft beers by HPLC-ESI-MS/MS. Food Chem..

[30] Huang X., Li J., Li M., Huang J., Jiang X., Fu H., Wu J., Bao M., Wang S., Zhang M. (2021). Polyphenol-Enriched Extracts from Trapa acornis Husks Inhibit Her2-Positive SK-BR-3 Breast Cancer Cell Proliferation and In Vivo Tumor Angiogenesis. Nutr. Cancer.

[31] Gruz J., Novák O., Strnad M. (2008). Rapid analysis of phenolic acids in beverages by UPLC–MS/MS. Food Chem..

[32] Zhang G., Chen S., Zhou W., Meng J., Deng K., Zhou H., Hu N., Suo Y. (2018). Rapid qualitative and quantitative analyses of eighteen phenolic compounds from Lycium ruthenicum Murray by UPLC-Q-Orbitrap MS and their antioxidant activity. Food Chem..

[33] Zhou Z.-M., Yan D.-M., Wang Y.-K., Zhang T., Xiao X.-R., Dai M.-Y., Zhang S.-W., Liu H.-N., Li F. (2021). Discovery of quality markers in Rubus chingii Hu using UPLC-ESI-QTOF-MS. J. Pharm. Biomed. Anal..

[34] Zan T., Piao L., Wei Y., Gu Y., Liu B., Jiang D. (2018). Simultaneous determination and pharmacokinetic study of three flavonoid glycosides in rat plasma by LC–MS/MS after oral administration of Rubus chingii Hu extract. Biomed. Chromatogr..

[35] Forli S., Huey R., Pique M.E., Sanner M.F., Goodsell D.S., Olson A.J. (2016). Computational protein–ligand docking and virtual drug screening with the AutoDock suite. Nat. Protoc..

[36] Lu S.-H., Wu J.W., Liu H.-L., Zhao J.-H., Liu K.-T., Chuang C.-K., Lin H.-Y., Tsai W.-B., Ho Y. (2011). The discovery of potential acetylcholinesterase inhibitors: A combination of pharmacophore modeling, virtual screening, and molecular docking studies. J. Biomed. Sci..

[37] Shi X., Yu W., Yang T., Liu W., Zhao Y., Sun Y., Chai L., Gao Y., Dong B., Zhu L. (2016). Panax notoginseng saponins provide neuroprotection by regulating NgR1/RhoA/ROCK2 pathway expression, in vitro and in vivo. J. Ethnopharmacol..

[38] Bussi C., Peralta Ramos J.M., Arroyo D.S., Gaviglio E.A., Gallea J.I., Wang J.M., Celej M.S., Iribarren P. (2017). Autophagy down regulates pro-inflammatory mediators in BV2 microglial cells and rescues both LPS and alpha-synuclein induced neuronal cell death. Sci. Rep..

[39] Liu Y., Song M., Che T., Bravo D., Pettigrew J. (2012). Anti-inflammatory effects of several plant extracts on porcine alveolar macrophages in vitro. J. Anim. Sci..

[40] Daniel D., Ana R., Jeremy P., Massimiliano T., Gina B., Alan C. (2013). Dietary (poly) phenolics in human health: Structures, bioavaility, and evidence of protective effects against chronic diseases. Antioxid Redox Signal..

[41] Ríos Cañavate J.L., Giner Pons R.M., Marín Vázquez M., Recio Iglesias M.C. (2018). A pharmacological update of ellagic acid. Planta Med..

[42] García-Niño W.R., Zazueta C. (2015). Ellagic acid: Pharmacological activities and molecular mechanisms involved in liver protection. Pharmacol. Res..

[43] Chen L., Wang X., Lu W., Wu X., Li J. (2016). Molecular imprinting: Perspectives and applications. Chem. Soc. Rev..

[44] Gokulakrishnan S., Arthanareeswaran G., Gnanasekaran G., László Z., Veréb G., Kertész S., Taweepreda W. (2022). Advanced extraction and separation approaches for the recovery of dietary flavonoids from plant biomass: A review. Biomass Convers. Biorefinery.

[45] Baker Z.K., Sardari S. (2021). Molecularly imprinted polymer (MIP) applications in natural product studies based on medicinal plant and secondary metabolite analysis. Iran. Biomed. J..

[46] Kaewkaen P., Tong-Un T., Wattanathorn J., Muchimapura S., Kaewrueng W., Wongcharoenwanakit S. (2012). Mulberry fruit extract protects against memory impairment and hippocampal damage in animal model of vascular dementia. Evid. -Based Complement. Altern. Med..

[47] Najafi M., Yousefi Y., Rafati A. (2012). Synthesis, characterization and adsorption studies of several heavy metal ions on amino-functionalized silica nano hollow sphere and silica gel. Sep. Purif. Technol..

[48] Baggiani C., Baravalle P., Giovannoli C., Anfossi L., Giraudi G. (2010). Molecularly imprinted polymers for corticosteroids: Analysis of binding selectivity. Biosens. Bioelectron..

[49] Bulani V.D., Kothavade P.S., Nagmoti D.M., Kundaikar H.S., Degani M.S., Juvekar A.R. (2015). Characterisation and anti-inflammatory evaluation of the inclusion complex of ellagic acid with hydroxypropyl-β-cyclodextrin. J. Incl. Phenom. Macrocycl. Chem..

[50] Foroughirad S., Haddadi-Asl V., Khosravi A., Salami-Kalajahi M. (2020). Synthesis of magnetic nanoparticles-decorated halloysite nanotubes/poly ([2-(acryloyloxy) ethyl] trimethylammonium chloride) hybrid nanoparticles for removal of Sunset Yellow from water. J. Polym. Res..

[51] Chowdhury P., Mondal P., Roy K. (2011). Synthesis of polyaniline nanoparticle grafted silica gel and study of its Cr (VI) binding property. J. Appl. Polym. Sci..

[52] Miura C., Li H., Matsunaga H., Haginaka J. (2015). Molecularly imprinted polymer for chlorogenic acid by modified precipitation polymerization and its application to extraction of chlorogenic acid from Eucommia ulmodies leaves. J. Pharm. Biomed. Anal..

[53] Cho J.-Y., Kim S.-J., Lee H.-J., Kim J.-Y., Lym I.-J., Kang S.-K., Park K.-H., Moon J.-H. (2011). Isolation and identification of low molecular volatile compounds from ethyl acetate layer of Korean black raspberry (Rubus coreanus Miq.) wine. Korean J. Food Sci. Technol..

[54] Cho J.-Y., Yoon I., Jung D.-H., Hyun S.H., Lee K.-H., Moon J.-H., Park K.-H. (2012). Jaboticabin and flavonoids from the ripened fruit of black rasberry (Rubus coreanum). Food Sci. Biotechnol..

[55] Zhi K., Wang L., Zhang Y., Jiang Y., Zhang L., Yasin A. (2018). Influence of size and shape of silica supports on the sol–gel surface molecularly imprinted polymers for selective adsorption of gossypol. Materials.

[56] Bortolato A., Fanton M., Mason J.S., Moro S. (2013). Molecular docking methodologies. Biomolecular Simulations.

[57] Tomaselli S., La Vitola P., Pagano K., Brandi E., Santamaria G., Galante D., D’Arrigo C., Moni L., Lambruschini C., Banfi L. (2019). Biophysical and in vivo studies identify a new natural-based polyphenol, counteracting Aβ oligomerization in vitro and Aβ oligomer-mediated memory impairment and neuroinflammation in an acute mouse model of Alzheimer’s disease. ACS Chem. Neurosci..

[58] Windsor P.K., Plassmeyer S.P., Mattock D.S., Bradfield J.C., Choi E.Y., Miller III B.R., Han B.H. (2021). Biflavonoid-induced disruption of hydrogen bonds leads to amyloid-β disaggregation. Int. J. Mol. Sci..

[59] Wang Y., Xu W., Yan Z., Zhao W., Mi J., Li J., Yan H. (2018). Metformin induces autophagy and G0/G1 phase cell cycle arrest in myeloma by targeting the AMPK/mTORC1 and mTORC2 pathways. J. Exp. Clin. Cancer Res. CR.

[60] Lutz M.B., Suri R.M., Niimi M., Ogilvie A.L., Kukutsch N.A., Rößner S., Schuler G., Austyn J.M. (2000). Immature dendritic cells generated with low doses of GM-CSF in the absence of IL-4 are maturation resistant and prolong allograft survival in vivo. Eur. J. Immunol..

[61] Bai L., Hu H., Zhang W., Fu J., Lu Z., Liu M., Jiang H., Zhang L., Chen Q., Tan P. (2012). Amine/acid catalyzed synthesis of a new silica-aminomethyl pyridine material as a selective adsorbent of copper. J. Mater. Chem..

[62] Wada L., Ou B. (2002). Antioxidant activity and phenolic content of Oregon caneberries. J. Agric. Food Chem..

[63] Bae J.Y., Choi J.S., Kang S.W., Lee Y.J., Park J., Kang Y.H. (2010). Dietary compound ellagic acid alleviates skin wrinkle and inflammation induced by UV-B irradiation. Exp. Dermatol..

[64] Zheng P., Zhang B., Luo Z., Du W., Guo P., Zhou Y., Chang R., Chang C., Fu Q. (2018). Facile preparation of polydopamine-coated imprinted polymers on the surface of SiO2 for estrone capture in milk samples. J. Sep. Sci..

[65] Cormack P.A., Elorza A.Z. (2004). Molecularly imprinted polymers: Synthesis and characterisation. J. Chromatogr. B.

[66] Hidayat A.R.P., Sulistiono D.O., Murwani I.K., Endrawati B.F., Fansuri H., Zulfa L.L., Ediati R. (2021). Linear and nonlinear isotherm, kinetic and thermodynamic behavior of methyl orange adsorption using modulated Al2O3@ UiO-66 via acetic acid. J. Environ. Chem. Eng..

