# Supplementary material for: Isolation of Phenolic Compounds from Raspberry Based on Molecular Imprinting Techniques and Investigation of Their Anti-Alzheimer’s Disease Properties
Source: Molecules. 2022 Oct 14;27(20):6893. doi: 10.3390/molecules27206893 (PMC9611113; doi:10.3390/molecules27206893)
Supplement: Supplementary file 1 [file molecules-27-06893-s001.zip › molecules-1941190-supplementary.pdf]

Isolation of Phenolic Compounds from Raspberry Based on Molecular Imprinting Techniques and Investigation of Their Anti-Alzheimer's Disease Properties

Qian Wu [1], Abid Naeem [2], Jiamei Zou [1], Chengqun Yu [2], Yingjie Wang [1], Jingbin Chen [3] and Yuhui Ping [1,*]

[1] College of Pharmacy, Jiangxi University of Traditional Chinese Medicine, 1688 Meiling Avenue, Nanchang 330004, China
[2] Key Laboratory of Modern Preparation of Traditional Chinese Medicines, Ministry of Education, Jiangxi University of Chinese Medicine, 1688 Meiling Avenue, Nanchang 330004, China
[3] Key Laboratory of Depression Animal Model Based on TCM Syndrome, Jiangxi Administration of Traditional Chinese Medicine, Key Laboratory of TCM for Prevention and Treatment of Brain Diseases with Cognitive Impairment, Jiangxi University of Chinese Medicine, 1688 Meiling Road, Nanchang 330006, China
* Correspondence: 20030772@jxutcm.edu.cn

Table S1. Molecular docking results of the 6 phenolic compounds with target proteins of AD.

Small molecule	Proteins	LibDock Score
Ellagic acid	AchE	93.3618
	α7nAChR	80.9835
	Muscarnic M1	95.8166
	GSK3β	109.773
	GABAA	70.0911
	AMPA2	89.6029
	PDE4A	97.6808
	PDE4B	75.7554
Kaempferol-3-o-rutoside	AchE	79.1189
	α7nAChR	109.245
	γsecretase	95.5422
	4MR7	84.4924
	GABAA	80.1600
	PDE4B	80.5874
Gallic acid	AchE	67.8963
	α7nAChR	74.7093
	Muscarnic M1	68.3347
	γsecretase	84.1079
	GSK3β	79.6439
	CDK5	86.4328
	PDE4B	55.4700

	AMPA2	69.0979
	MAOB	83.2873
	PDE4A	79.4704
	PDE4B	66.2703
	Sigmal receptor	83.7129
	H_3R	65.9726
Vanillic acid	AchE	67.0157
	α7nAChR	61.1434
	Muscarnic M1	55.3300
	γsecretase	80.8800
	GSK3β	73.3396
	CDK5	81.7991
	GABAA	67.9961
	AMPA2	56.6690
	MAOB	69.3328
	PDE4A	75.4865
	PDE4B	77.7366
	H_3R	61.7985
Ferulic acid	AchE	68.5100
	α4nAChR	72.7063
	α7nAChR	70.4978
	Muscarnic M1	75.1111
	γsecretase	97.2015
	GSK3β	87.6039
	CDK5	82.6024
	GRIN2B	76.1808
	GABBR2	71.6768
	GABBA	54.1716
	AMPA2	64.5998
	MAOB	84.1904
	PDE4A	84.7020
	PDE4B	82.6732
	H_3R	80.2541
Tiliroside	AchE	124.193

α4nAChR	132.099
α7nAChR	122.9310
γsecretase	117.8800
GSK3β	126.7130
GABAA	112.0150
GABAB	84.8216
AMPA2	89.4694
PDE4A	134.3560
PDE4B	115.9470
